# A Beginner’s Guide for Using Bone in Marine Mammal Research

**DOI:** 10.1093/icb/icag145

**Published:** 2026-08-06

**Authors:** Patrick Charapata, Casey T Clark, Emily S Sperou, Brooke Morris, Lara Horstmann, Nicole Misarti

**Affiliations:** Center for Species Survival, Georgia Aquarium, 225 Baker St NW, Atlanta, GA 30313, USA; Washington Department of Fish and Wildlife, Wildlife Science Division, Olympia, WA 98501, USA; Natural Resources Science, College of the Environment and Live Sciences, University of Rhode Island, Kingston, RI 02881, USA; Department of Biology, Baylor University, Waco, TX 76798, USA; College of Fisheries and Ocean Sciences, University of Alaska Fairbanks, Fairbanks, AK 99775, USA; Institute of Northern Engineering, University of Alaska Fairbanks, Fairbanks, AK 99775, USA

## Abstract

Climate change has drastically altered ecosystems world-wide, making animal population data from before the modern era (<1950 CE) essential for establishing accurate baselines against which these changes can be measured. As a mineralized tissue composed of organic and inorganic materials, bone can archive ecological, physiological, and genetic data. Zooarchaeologists and archaeologists have developed extensive research disciplines using bones to study animal population changes over broad time periods; however, these approaches are underutilized in studies of marine mammal species. Here, we present a practical beginner’s guide for marine mammal biologists who are interested in using bone as a tool to perform retrospective studies on animal physiology, ecology, genetics, and conservation. Specifically, we give a brief overview of bone biology and the biomarkers preserved in different bone components. We provide suggested methods and best practices for destructive sampling and maximizing analyte data from small bone sample sizes, while also noting methodological limitations. We present a case study that highlights the use of bone to collect ecological, physiological, genetic, and biometric data for the Pacific walrus. Finally, we provide a conceptual guide that helps researchers identify the best methods for investigating their research question(s) of interest. We suggest bone is a critical tissue to address questions related to how adaptable marine mammal populations are to their rapidly changing environments in the postindustrial era.

## Purpose of study and scope

Human-induced climate change is impacting terrestrial and aquatic ecosystems worldwide (IPCC 2022). The period between 1750–1900 common era (CE) is often used as a pre-industrial reference point, with 1950 CE treated as the start of temperature increases associated with human industrialization (IPCC 2022). For decades, researchers have studied how wildlife populations will adapt or perish in response to environmental changes (Dawson et al. 2011; Glick et al. 2011; Thurman et al. 2020). It will be critical to expand the toolkit of researchers to address shifting population baselines as changes in climate and ecosystems persist (Soga and Gaston 2018).

Few animal tissues are preserved for long periods in nature or without cold storage, making bone one of the most viable options to assess the health of animals over extensive time periods. The purpose of this beginner’s guide is to introduce biologists to the unique properties and biological data in bone for addressing wildlife conservation questions. This manuscript focuses on the application of bone to study marine mammals, but much of this information applies to other vertebrates. The use of bone to study wildlife conservation and evolution has been well-developed in the paleoecology, archaeology, and zooarchaeology disciplines. Additionally, the medical field is instrumental for understanding mechanisms of bone physiology and biology in humans and other animals, which can be applied to marine mammal research. This guide is not intended to be a comprehensive review of the literature associated with these fields. Instead, we reference targeted manuscripts, textbooks, or other broad reviews and their sources for readers to consult further.

In this manuscript, we give a brief overview of bone biology, the data obtained from nondestructive and destructive analyses of bone, introductory information on performing bone studies, and a blueprint of suggested methods for destructive sampling and maximizing data produced from small bone sample sizes, while also noting key caveats and limitations. We provide a case study that highlights the use of bone to collect complementary ecological, physiological, genetic, and biometric data for a marine mammal species. Finally, we develop a conceptual framework that helps researchers identify the best methods to investigate their research question(s) along with future research suggestions and conclusions.

## Introduction

### Bone composition and homeostasis

Bone is a hard connective tissue that is a major component of the skeletal system and, mechanically, provides structures for muscle and tendon attachments allowing movement, while also providing protection for internal organs. Bones can be classified into four main categories based on their shape: long bones, short bones, flat bones, and irregular bones (Clarke 2008).

**Fig. 1:**
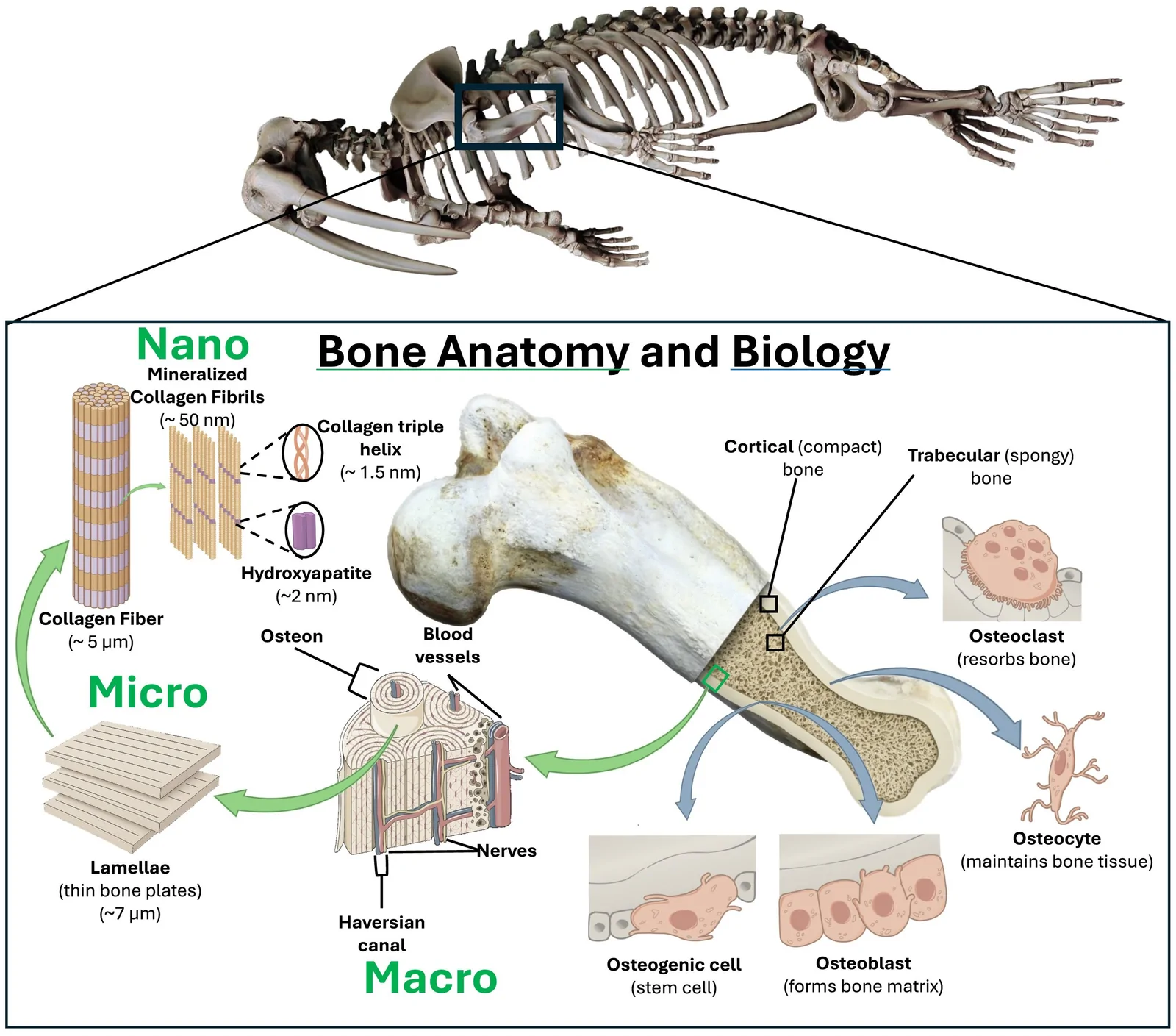
General anatomy and biology of vertebrate bone. Different bone compartments are depicted in a cross-section of a humerus from a walrus skeleton (UAM Mamm: 14793) along with bone cells responsible for tissue turnover. Illustrations of bone cells and “macro” bone are adapted from Biga et al. (2025) under CC BY-SA 4.0 found here. Lamellae and cross-section of bone illustrations were generated using artificial intelligence (e.g., Microsoft Copilot) while other illustrations are from BioRender. Image credits for walrus skeleton and humerus: Idaho Virtual Museum, Idaho Museum of Natural History.

Internally, bones contain organic and inorganic materials, are well-vascularized, and have specific cell types to help with bone physiology (Tomlinson and Silva 2013). The inorganic and organic composition varies to allow for strength, elasticity, and flexibility as required by each bone’s function (Clarke 2008). The innermost portion of bone is called trabecular bone, also referred to as the “spongy” bone, because in addition to hard bone tissue it contains the bone marrow and major vessels for transport of biomarkers of research interest (Fig. 1). Cortical bone is “compact” and provides structural support, protection, and acts as a reservoir of minerals, lipids, and other biomarkers within its matrix (Honda et al. 1984; Black and Tadros 2020). It is also vascularized, allowing transport of various biomarkers for bone health and to maintain bone homeostasis (Black and Tadros 2020).

#### Bone remodeling

Bone is a dynamic tissue that undergoes continuous remodeling or turnover processes to maintain homeostasis which is broadly regulated through coordination among bone cells; osteocytes, osteoblasts, and osteoclasts (Usha and Nandeesh 2012). Osteocytes are embedded in the mineralized bone matrix and compose approximately 90–95% of cells in bone (Fig. 1) (Capulli et al. 2014). Osteocytes have a weblike structure that forms a network of cells capable of detecting mechanical stress signals, such as microfractures or bone thinning (Wawrzyniak and Balawender 2022). Once osteocytes identify areas requiring bone repair, osteoclasts (bone resorbing cells) and osteoblasts (bone forming cells) work together as basic multicellular units (BMUs) to absorb old and form new bone, respectively (Wawrzyniak and Balawender 2022). Osteoclasts deconstruct the old bone matrix and then absorb the material through phagocytosis (Wawrzyniak and Balawender 2022). Osteoblasts then begin to synthesize Type I collagen proteins, noncollagen proteins, and proteoglycans, into an organic matrix termed an osteoid (Capulli et al. 2014). The organic matrix is the foundation and site of biomineralization; the process of incorporating inorganic mineral crystal structures, such as hydroxyapatite [Ca_10_(PO_4_)6(OH)_2_], into newly formed bone tissue (Sharma et al. 2021). Ions in hydroxyapatite can be substituted with other ions (e.g., Mg, Na, K for Ca and carbonates for phosphates) forming bioapatite (Kuczumow et al. 2022). The result of bone remodeling process within cortical bone is an osteon, which includes layers of bone encapsulated by cement lines around a central “Haversian canal” containing blood vessels and nerves (Fig. 1). The Haversian canals are not always formed when remodeling endocortical or trabecular bone (Allen and Burr 2019).

In humans, the breakdown and formation of new bone can take up to 6 months, while mineralization can continue for up to a year (Allen and Burr 2019). Remodeling is not consistent across elements (Allen and Burr 2019), with complete bone and bone collagen turnover in humans ranging between 8 and 28 years (Quinn et al. 2025).

#### Marine mammal bone specifications

Marine mammals are defined by their reliance on ocean resources and include three main clades; cetaceans, pinnipeds, and sirenians, but also include sea otters and polar bears. Detailed reviews of marine mammal evolution can be found in (Heyning and Lento 2009; Davis 2019a, 2019b). Generally, marine mammal bones are more mineralized and denser compared to terrestrial mammals of comparable size to aid in buoyancy control and movement in a marine environment (Domning and De Buffrénil 1991; Gray et al. 2007; Amson and Bibi 2021). For example, increased bone density is critical in some marine mammals, e.g., walruses (*Odobenus rosmarus*) and Sirenia, to offset positive buoyancy of fat and digestive gases (Crowell et al. 2020). Cetaceans are completely aquatic mammals and have evolved a diverse array of bone adaptations, such as increased amounts of trabecular bone in large whales for lipid storage (George et al. 2016) and increased flexibility for intense pressures experienced by diving whales (Sun et al. 2019). In contrast, smaller more dynamic cetaceans (e.g., delphinidae) have greater bone density for dealing with acrobatics and fast-swimming foraging behaviors (Sun et al. 2019). Bone density adaptations are achieved through changing the microstructure of different bones most likely through the evolutionary selection of adaptive bone remodeling mechanisms (Gray et al. 2007; de Buffrénil et al. 2010). Marine mammal bones contain variable amounts of water, inorganic materials [minerals, bioapatite], proteins [mostly collagens], and lipids among bone elements and across taxa (Tont et al. 1977; Newsome et al. 2010; Higgs et al. 2011; Bas et al. 2020).

Remodeling rates provide important contextual information when measuring compounds or elements of interest in bone, but they are mostly unknown for marine mammals. Some bones annually deposit alternating light and dark colored layers of tissue commonly referred to as growth layer groups (or GLGs), including ear bones, mandibles, periotic dome of the skull, and ribs; however, GLGs are eventually lost to remodeling and bone absorption making them unreliable for aging past certain years for some species (Laws 1960; Garlich-miller et al. 1993; Read et al. 2018; Lonati et al. 2021). The application of bone turnover rates from humans to marine mammals has been attempted (Charapata et al. 2018) but requires further study.

## Conservation data obtained from bones

Bone provides a wealth of data that can inform marine mammal biology, physiology, ecology, evolution, and conservation. Here, we provide a brief description of analyses, both nondestructive and destructive, to obtain various physiological, ecological, contaminant, and genetic data from vertebrate bone, including those of marine mammals. After each subsection, we discuss the recommended method for analysis, while noting major limitations as necessary.

### Non–destructive analyses

Non–destructive analyses generally focus on measurements or scans of the physical structure of bone specimens, which is most commonly used to provide insight into stressors and disease occurrence. These approaches can thus address research questions related to the frequency and intensity of stressors on marine mammal populations, as well as the prevalence of diseases, and can link measurements of other analytes such as heavy metals or contaminants to functional changes in animal physiology.

#### Developmental stability

Developmental stability is defined as the ability of an organism to buffer against genetic or environmental disturbances encountered during development to produce the genetically predetermined phenotype (Sánchez-Chardi et al. 2013). Reduced developmental stability can manifest as an increase in bilateral asymmetry in the body. These random deviations from symmetry are known as fluctuating asymmetry and are a key measure of developmental stability. Overall, more symmetrical individuals show higher reproductive success and survival (Møller 1997) . Some stressors known to cause fluctuating asymmetry are disease, contaminant exposure, genetic mutations, inbreeding, and nutritional stress (Swaddle and Witter 1994).

##### Methods for developmental stability

Methods to investigate fluctuating asymmetry in bilateral animals, such as mammals, typically involve scanning and comparing landmarks (matching points) on the right and left side of the body (Graham et al. 2010). The greatest degree of asymmetry can be expected in landmarks with functions in feeding, echolocation, and other biomechanical factors (Laeta et al. 2023; Manuta et al. 2026). Scans can be completed by computed tomography (CT) or other 2D/3D imaging techniques followed by statistical analyses, such as Procrustes superimposition to measure deviations between matching landmarks (Klingenberg et al. 2002). For example, using > 30 morphometric measurements of landmarks on the left and right side of harbor seal (*Phoca vitulina*) skulls from the North Sea, researchers demonstrated higher developmental instability (i.e., less symmetry) following exposure to pollutants after World War II (Schandorff 1997). Even basic measurements like basal circumference differences between walrus tusks can provide insight into stress over time (MacCracken and Benter 2016). However, a major limitation of manually measuring landmarks is a lack of statistical power for detecting small deviations from natural variability between landmarks (Schandorff 1997; MacCracken and Benter 2016). Advances in technology (e.g., manual high-resolution 3D scanners) have led to more sophisticated imaging of specimens and subsequent landmark and statistical analyses (Coombs et al. 2020; Manuta et al. 2026). These advanced methods have led to robust studies of describing where asymmetry evolved from in cetaceans (Coombs et al. 2020), but beginners may want to inquire about collaborating with an appropriate expert on their species of interest.

#### Bone mineral density

Changes to bone mineral density are another valuable indicator of chronic stress on an individual. Reduction in bone mineral density has been described in a wide variety of vertebrates in response to both chronic and acute stressors (Suarez-Bregua et al. 2018). For example, male polar bears from East Greenland showed a significant decline in bone mineral density from 1892–2015, with skulls sampled from a period of elevated environmental pollution (1966–2015) having an increased prevalence of osteopenia, a precursor to osteoporosis (Daugaard-Petersen et al. 2018). Baltic pinnipeds demonstrated similar bone density declines in response to pollutants (Sonne et al. 2020).

##### Methods for bone mineral density

Bone density can be assessed minimally invasively on archived bone material via peripheral dual-energy X-ray absorptiometry (DEXA) or ultrasound (Sonne et al. 2004; Kaufman et al. 2007; Powell et al. 2021). A DEXA scan is the most common and simple way to calculate bone mineral density, measured in grams of hydroxyapatite per square centimeter of bone. This DEXA method has been implemented on polar bear and bottlenose dolphin bones without any destruction to the specimen (Sonne et al. 2004; Powell et al. 2021).

#### Bone lesions

Bone lesions can be informative in assessing health of individuals. Lesions can be caused by exposure to some infectious agents, age-related changes, and barotrauma. Osteomyelitis (inflammation of the bone) in marine mammals is typically bacterial in origin, often caused by organisms such as *Staphylococcus* and *Brucella* spp. (Goertz et al. 2011; San Martín et al. 2016). Osteoarthritis (degeneration of the articular surface of joints) has also been linked to infections like *Brucella* spp. (Nganvongpanit et al. 2017), though its prevalence in marine mammals is typically lower than in their terrestrial counterparts as buoyancy reduces stress on the joints. Within marine mammals, dugongs (*Dugong dugon*) and dolphins showed the highest osteoarthritis prevalence (Turnbull and Cowan 1999; Nganvongpanit et al. 2017). Osteoarthritis appears to be related to age in bottlenose dolphins (*Tursiops truncatus*) (Barratclough et al. 2023). Osteonecrosis resulting from barotrauma (i.e., decompression sickness) has been described in several deep-diving cetacean species (Moore and Early 2004; Consoli et al. 2025). This is especially interesting as these chronic bone lesions imply repeated exposure to dysbarism followed by recovery. Osteonecrosis can also be caused by parasitic infections, e.g., *Crassicausa* spp. (van Bressem et al. 2006). Finally, trauma, as result of vessel strikes, is a leading cause of fatal bone fractures (Schoeman et al. 2020), but there is also evidence that some of these encounters have been survived (Daume et al. 2023).

##### Methods for bone lesions

**Fig. 2:**
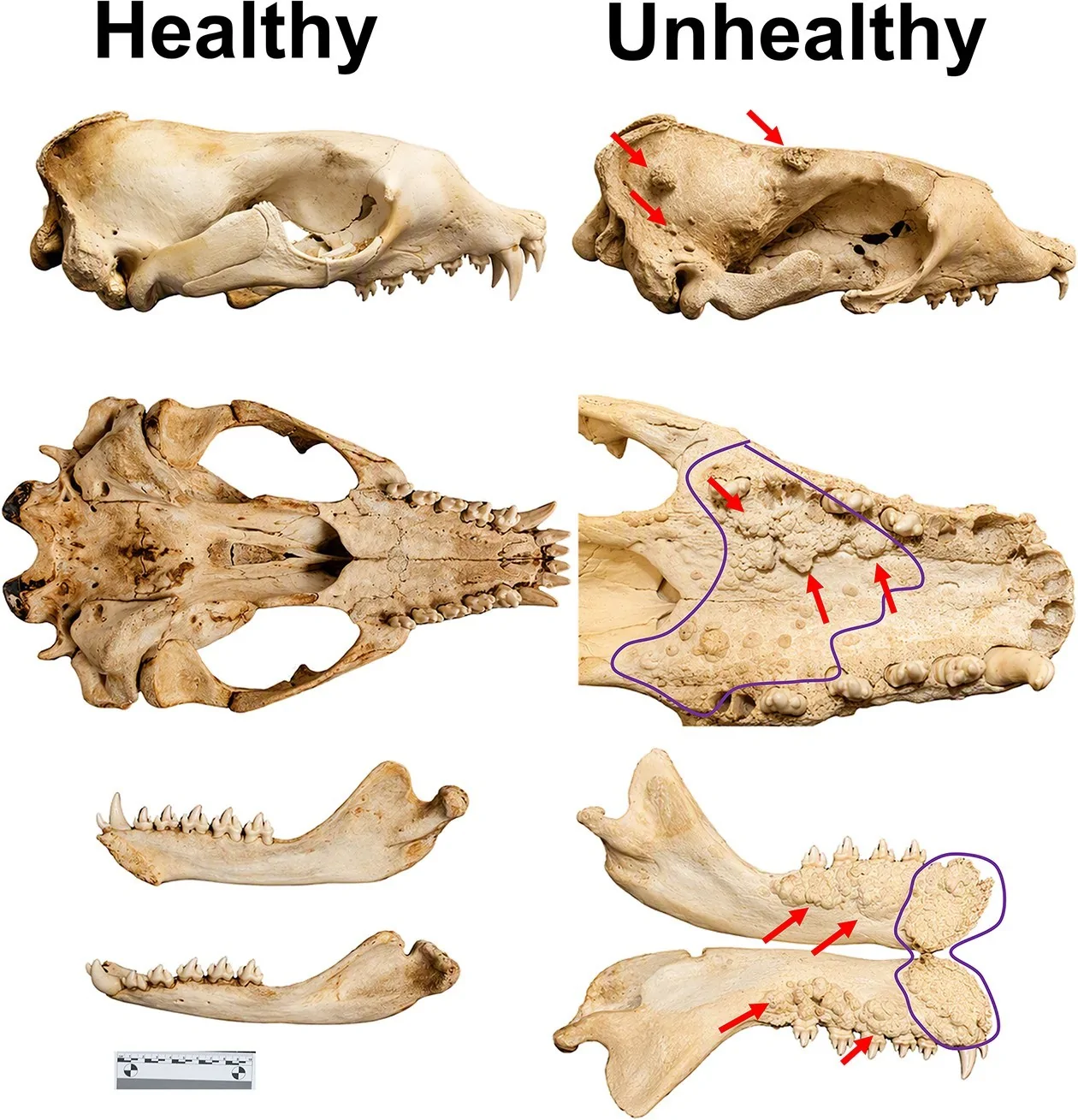
Comparative macroscopic analysis of healthy and unhealthy leopard seal skulls and mandibles. Healthy (specimens top to bottom: SMITH: MAMM: 550359, SMITH: MAMM: 15758, and SMITH: MAMM:15758) and unhealthy (specimens top to bottom: SMITH: MAMM: 606165, SMITH: MAMM: 277255, SMITH: MAMM: 277255) leopard seal skull and mandibles housed at the Smithsonian Institution National Museum of Natural History. Right panel of unhealthy bones include arrows to identify abnormal bone growths, while circled areas indicate general area of diseased bone compared to healthy skulls and mandibles on the left.

Bone features, such as Harris lines (i.e., growth arrest lines) in long bones, can be visualized in CT scan and X-rays, as well as histologically (Kulus et al. 2024). Other bone abnormalities and lesions can be observed both microscopically and macroscopically. Here, we provide an example of how macroscopic lesions in leopard seal (*Hydrurga leptonyx*) skulls can be detected and comparatively studied with healthy bones using noninvasive methods (Fig. 2); however, thorough measurements, high-quality photos, and statistical analysis are required for a rigorous macroscopic analysis (see Nganvongpanit et al. 2017).

### Destructive analyses

#### Physiological

##### Hormones

Hormones, specifically steroids, have been measured in bones of various vertebrates. Steroids are derived from cholesterol, a lipid that is deposited and can remain stable in bones for millennia (Evershed et al. 1995; Tintut and Demer 2014) making them ideal targets for assessing animal physiology from preindustrial to modern time periods. Steroid hormones control reproductive cycles (progesterone, estradiol, and testosterone) and the physiological stress response (glucocorticoids, including cortisol), whereas thyroid hormones help regulate metabolism (Norris 1997). Measurements of hormones in marine mammal bones can be used to address research questions related to reproductive physiology, life history, and development, as well as individual- and population-level responses to stressors.

**Fig. 3:**
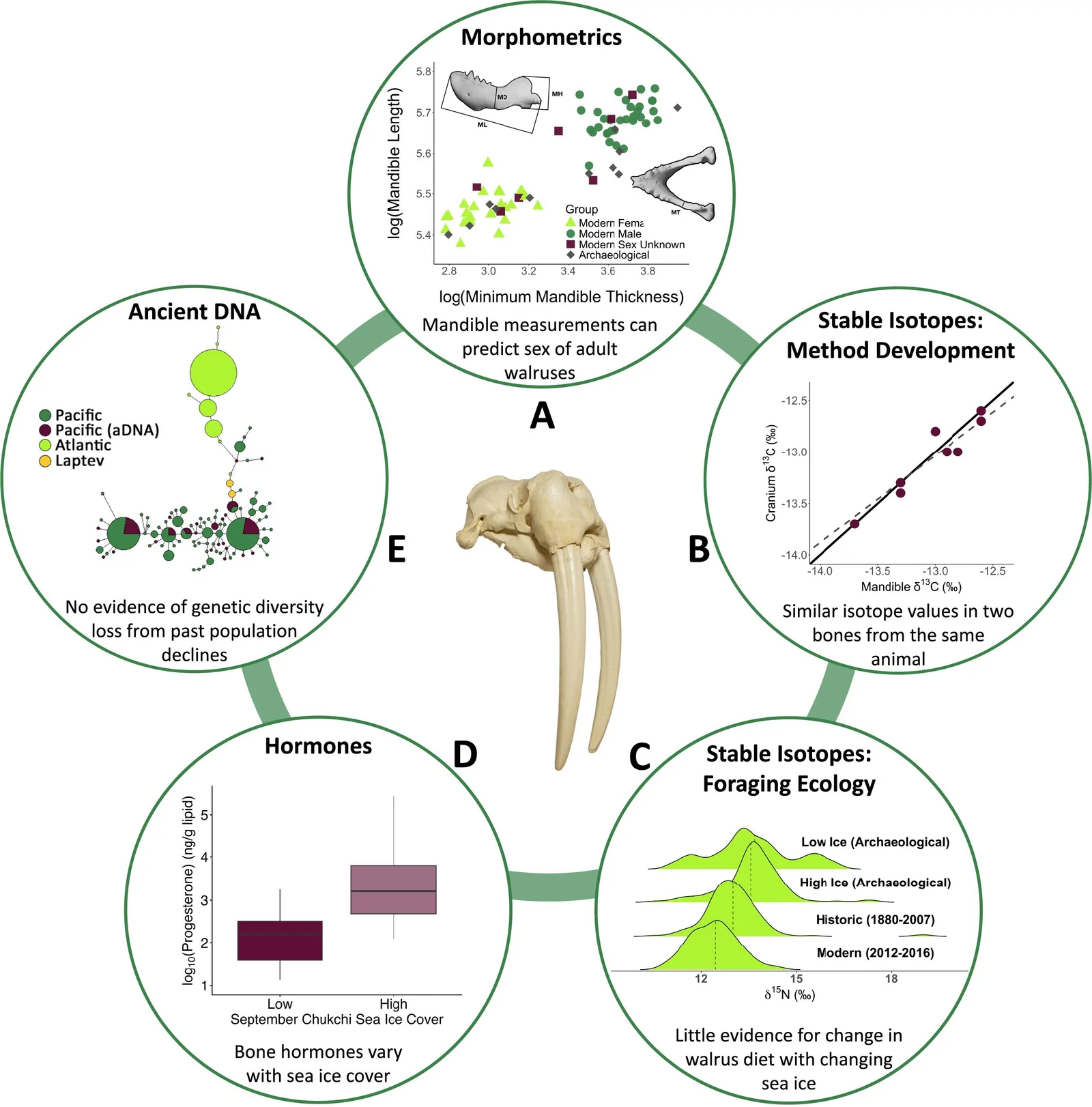
Key results from a multifaceted study of Pacific walrus bone parameters. Analyses include: (A) morphometrics (Taylor et al. 2020); (B) and (C) stable isotopes (Clark et al. 2017, 2019a); (D) hormones (Charapata et al. 2021); and (E) ancient DNA (Mills et al. 2024).

Steroids (progesterone, testosterone, estradiol, and cortisol) have been quantified in archaeological (defined as excavated by archaeological standards, with preserved contextual information), historical, and modern cortical bone of one marine mammal, the Pacific walrus (*Odobenus rosmarus divergens*) (Charapata et al. 2018) (Fig. 3). Hormones in walrus cortical bone may represent an accumulated average hormone signature over many years (progesterone, testosterone, and cortisol) or a year (estradiol) of the animals’ life, because of the lengthy walrus reproductive cycle (females up to 1.5 years) (Fay 1982; Larsen Tempel and Atkinson 2020) and the relatively slow cortical bone turnover rate (Clarke 2008; Charapata et al. 2018). Bone hormones correlated with estimated walrus population abundance and sea ice extent, suggesting that bone hormones can help assess physiological resiliency of walruses to climate change (Charapata et al. 2021).

###### Methods for hormones

A modest amount of sample processing is required to measure hormones in bone. We recommend pulverizing cortical bone into a powder (target: 250 mg) to maximize the surface area: volume ratio when the sample interacts with the organic solvent (e.g., 100% methanol or methyl-diether) during lipid extraction. Lipid extractions can vary from a 24-h solvent extraction using a tube rotator to more robust extractions, such as a Soxtec (modified Soxhlet; Anderson 2004) that adds heat to extract additional lipids. The addition of heat may impact the quality of bone collagen, if lipid-extracted bone powder is planned to undergo a collagen extraction for additional analyses (see below). Although not yet tested in marine mammal bone, pulverizing bone in a solvent and adding a solid-phase extraction step improved hormone detection in bone samples (Colldén et al. 2022; Unger et al. 2023) compared to solid–liquid extraction only (Charapata et al. 2018). Lipid content (weight of bone powder before lipid extraction minus weight after extraction) should be recorded for each sample and can vary over long periods in bones (decades to centuries) due to leaching or degradation. It is thus recommended hormone concentrations are corrected by their lipid content (ng of hormone/g of lipid); however, if comparing paired hormone concentrations measured in different matrices (e.g., blubber [ng/g], serum [ng/mL]) traditional units (e.g., ng of hormone/g of bone) is acceptable (Charapata et al. 2018).

Hormones can be measured in sample extracts using mass spectrometry (MS, e.g., liquid chromatography tandem mass spectrometry [LC/MS/MS]) or immunoassay (IA, e.g., enzyme-linked immunosorbent assays [ELISAs]) techniques depending on availability of time and resources. If using MS, it is required to add isotopically labeled internal hormone standards to each sample prior to any extractions to account for hormone loss during extraction. Derivatization of samples may be required (Charapata et al. 2018; Unger et al. 2023) for higher sensitivity of low hormone content in bones; however, recent advances have allowed for the removal of this step (Schrader et al. 2026). Mass spectrometry has the benefit of measuring a suite of hormones in one analytical run; however, it requires a trained technician to run the samples and can be more costly compared to ELISAs. While ELISAs have not been used for marine mammal bone, results from other vertebrates suggest ELISAs reliably measure hormones in bone (Yarrow et al. 2010; Charapata et al. 2022), although LC/MS/MS may be required for archaeological bones due to their low hormone concentrations (Charapata et al. 2018; Schrader et al. 2026). A major benefit of ELISAs is that they can be easily run in most labs following a simple protocol from a commercial manufacturer. The primary drawback of ELISAs is that if researchers are targeting a suite of hormones, multiple ELISA kits must be used, requiring greater sample mass and additional lab time compared to MS methods.

Whether using MS or IA methods, analytical and physiological validations are required when quantifying hormones in bones of new species. Analytical validations (e.g., linearity, accuracy, matrix interference) verify the correct hormone is accurately quantified in a sample, while physiological validations establish hormones are biologically relevant.

##### Fatty acids

Fatty acid (FA) signature analysis has emerged as a powerful alternative to traditional dietary methods, offering a longer-term and more integrated record of prey consumption in marine mammals. FAs are preserved in bone and are used to investigate long-term biochemical and ecological patterns. Specifically, circulating lipids are incorporated into both bone marrow and mineralized tissues during growth and remodeling resulting in FA profiles of marine mammal bones providing long-term records of diet and energy metabolism (Stott et al. 1997; Ames et al. 2002; Rendina-Ruedy and Rosen 2020). Fatty acid analysis can be used to address research questions related to marine mammal foraging strategies, historical changes in food webs, and sex- or age-related differences in diet.

When assessing FAs in well-preserved or fresh marine mammal bones, most studies have focused on bones that are saturated with lipids, such as the zygomatic process (Ames et al. 2002), mandible (Grahl-Nielsen et al. 1993), and vertebrae (Guilminot et al. 2014; Liebenau et al. 2015). The anatomical location is critical to consider, as FAs can vary across the body. For example, FAs in the melon and jaw fats of odontocetes (toothed whales) often exhibit shorter average chain lengths than those in blubber of the same individual (Ackman et al. 1971). Furthermore, when dealing with bone, it is also essential to account for degradation dynamics, as Liebenau et al. (2015) demonstrated that even during the earliest stages of decay, a portion of bone-derived fatty acids are metabolized by microbes, influencing long-term preservation.

###### Methods for fatty acids

Fatty acid analysis of marine mammal bone follows a standard protocol. Briefly, a sample mass of 50 mg (modern bone) or 300–600 mg (archaeological bone) of cortical bone powder is required for FA analysis (Grahl-Nielsen et al. 1993). In certain instances (i.e., recently perished individual), pretreatment of bones is required to remove exogenous lipids from bone due to residual soft tissue (Colonese et al. 2015; Scott 2021). Lipids for FA analysis can be extracted using organic solvents (such as hexane) or other methods mentioned above. Once lipids are extracted, FAs are typically analyzed following methanolysis, where methanol and an acid or base catalyst convert complex lipids into fatty acid methyl esters for quantification by gas chromatography (GC; Guilminot et al. 2014; Liebenau et al. 2015). Application of these methods enabled successful extraction of FAs from degraded whale vertebrae (Guilminot et al. 2014) and revealed two distinct population FA profiles in harp seal mandibles, allowing differentiation between populations (Grahl-Nielsen et al. 1993). It is important to sample the same element to minimize variability in FA composition due to degradation over time and differences in FA composition among elements (Scott 2021). Further, pairing FAs with compound-specific stable isotope analysis may provide deeper insight into dietary sources of FAs than composition analysis alone (Colonese et al. 2015, 2017) although this has not been attempted with marine mammal bone.

#### Ecological

##### Bulk and compound-specific stable isotopes

Stable isotope analysis is a longstanding and well-developed field that uses ratios of heavy and light isotopes to provide information about a sample. This approach has been widely used in both the mineral and organic components of bone, including in marine mammals; however, the majority of marine mammal bone stable isotope studies has focused on bulk stable carbon (δ^13^C) and nitrogen (δ^15^N) isotopes (Newsome et al. 2010). Other stable isotopes less commonly studied in marine mammals include hydrogen, oxygen, and sulfur (e.g., Crawford et al. 2008; Szpak and Buckley 2020; Cani et al. 2023; Séon et al. 2024). Because bone studies may involve analysis of specimens from across broad time periods, the impact of anthropogenic input of CO_2_ on δ^13^C (called the Suess Effect) should be considered and corrected for when comparing samples before and after the beginning of industrialization (∼1850 CE) or across more than a handful of years within the industrialized era (Clark et al. 2021a). Stable isotope analysis can be used to address research questions related to marine mammal diet, movements, migration, population structure, physiology, and life history.

Bulk stable isotope analysis is the most common approach used for isotopic research of animals. This analysis measures the isotope ratios of an element or elements of interest within an entire sample, providing an average value that incorporates any variability within individual components of the sample (e.g., proteins, lipids, and minerals). Bone’s continuous, slow remodeling leads to similarly slow isotopic turnover rates, meaning stable isotope values measured in bone typically represent much longer timeframes (years to lifetime) than soft tissues, which usually provide information ranging from days to months (Riofrío-Lazo and Aurioles-Gamboa 2013). This slow turnover has advantages and disadvantages when used to address different research questions, as short-term variability is likely to become smoothed over by longer term or larger amplitude signals, which may persist in the bone for extended periods.

In contrast to bulk stable isotope analysis, compound-specific analysis focuses on measuring the stable isotope ratios of an element within a certain component of a sample, typically amino acids or fatty acids in a biological sample (Whiteman et al. 2019). This approach removes some of the variability and ambiguity that can come from analyzing multiple compounds simultaneously, as the isotope values of each may be driven by different factors and their relative proportions vary from sample to sample. A strength of this type of analysis is that if the drivers of isotope ratios of individual compounds are known (e.g., stable nitrogen isotope ratios of some amino acids are closely linked to trophic position), it allows for precise measurement of that signal while removing the inherent variability in bulk stable isotope analysis (Styring et al. 2010). However, compound specific stable isotope analysis is a newer approach and much remains to be learned about the specific processes that drive stable isotope ratios of individual compounds, particularly in marine mammals (Matthews et al. 2020). Only a handful of studies have applied compound specific stable isotope analysis to marine mammal bones, with most of these focusing on sea otters and all measuring C and/or N isotope values of amino acids derived from bone collagen (Matthews and Ferguson 2014; Elliott Smith et al. 2020, 2025; Feddern et al. 2022; Robinson et al. 2024).

##### Species identification (ZooMS)

Though unrelated to stable isotope analysis, a technique that can be paired with isotopic analysis of bone collagen is zooarchaeology by mass spectrometry (ZooMS; Buckley et al. 2009). This approach uses “fingerprinting” of peptides in bone collagen to identify or confirm the species from which the bone originated. This technique was developed to identify species of bones recovered from middens, and less frequently for bones in historical museum archives. While species is likely known for most contemporary specimens, ZooMS can be easily and relatively inexpensively conducted on collagen samples to determine or confirm the species from which the sample originated. ZooMS has been validated for use in marine mammals (Buckley et al. 2014), and it has found wide application to determine species for marine mammal bone specimens, particularly from large whales (e.g., Hufthammer et al. 2018; Szpak et al. 2018; Szpak and Buckley 2020; Wellman et al. 2025).

##### Methods for bulk and compound-specific stable isotope and ZooMS analysis

###### Collagen extractions (required for stable isotopes and ZooMS)

Most stable isotope and ZooMS analyses of bone have focused on the organic fraction (i.e., collagen), thus requiring collagen extraction. For standard bulk stable isotope analysis < 1 mg of bone collagen is typically required (e.g., Charapata and Trumble 2023), while 5–10 mg of collagen is required for compound-specific stable isotope analysis (Matthews and Ferguson 2014; Robinson et al. 2024) and 50 mg of unprocessed bone powder is used for collagen extractions during ZooMS (Buckley et al. 2014). This implies that only very small quantities of bone are used for collagen extractions. However, given the challenge of working with such small samples, variable collagen yield associated with sample quality, and the expected loss of some collagen during the extraction process, we recommend using larger samples of bone tissue for collagen extractions (≥ 250 mg). When possible, it may be worthwhile to extract more collagen than required to allow for analysis of replicate samples, reruns of failed samples, and long-term storage of collagen for future analysis.

Briefly, the process of collagen extraction involves removing lipids from a sample, dissolving the mineral fraction of the bone using a weak acid (typically dilute HCl), putting the collagen into solution through a process called gelatinization that breaks down the tightly woven collagen molecules (Fig. 1) into its smaller gelatin proteins, then filtering the resulting solution to remove any other impurities before freeze drying (Jørkov et al. 2007; Misarti et al. 2009; Tsutaya et al. 2017).

Collagen can be extracted from either powdered or solid bone samples, with pros and cons to each approach. Using powdered bone samples allows for more standardized sample processing and is faster, whereas extraction of collagen from solid bone samples allows the process to be adjusted to align with the preservation state and quality of each sample, thereby permitting more careful treatment of degraded samples (e.g., archaeological bone) and reducing loss of collagen during the extraction process (Jørkov et al. 2007; Tsutaya et al. 2017). These bone collagen extraction methods for solid bone were used in obtaining δ^15^N and δ^13^C data from degraded archaeological walrus bone (Fig. 3). However, if high quality bone samples are used, powdered bone can be used for the collagen extraction (Smith et al. 2020). In either case, sample quality is assessed by examining the atomic C: N ratio of the extracted collagen, with a ratio of 2.9–3.6 considered an indicator of good quality (Jørkov et al. 2007; Guiry and Szpak 2020). Lipids removed during collagen extraction can be preserved and used for other analyses, such as hormones or fatty acid analyses. If seeking to preserve lipids from samples being prepared for collagen extractions, sufficient sample mass should be used to ensure the success of both analyses. Lipid extraction processes that may degrade bone collagen, such as application of high temperatures for extended periods should be avoided.

###### Methods for bulk and compound specific stable isotope analysis

Bulk stable isotope analysis is the most straightforward and affordable approach to measure ratios of the most studied stable isotopes (δ^15^N and δ^13^C), with a wide variety of commercial and noncommercial labs available to conduct analyses. For this approach, a small subsample of bone collagen (∼0.2 mg) is enclosed in a foil capsule and combusted. An elemental analyzer coupled with an isotope ratio mass spectrometer then measures the ratio of targeted stable isotopes within the purified combustion products (typically CO_2_ for δ^13^C and N_2_ for δ^15^N). The resulting isotope ratios are then reported relative to the ratio in a globally accepted standard for that element, ensuring that results produced by laboratories around the world can be directly compared. Internal standards and blanks should be incorporated to assess accuracy and precision.

Compound-specific stable isotope analysis is a more recently developed approach that is more costly, time intensive, and requires additional sample preparation, but can address ecological or biological questions with more specificity than bulk stable isotope analysis. Whether measuring the stable isotope ratios of amino acids or fatty acids, these compounds must first undergo chemical derivatization, a process that volatilizes the molecules, thus allowing them to be separated and measured on a MS (e.g., Martinoia et al. 2025; Yamoah et al. 2025). The process of derivatization replaces hydrogen atoms in the target compounds following a stringent protocol that uses internal standards and chemicals with known isotope ratios to account for the impacts of these additions on the isotope ratios within the sample. The analysis provides isotope values for each amino acid or fatty acid targeted and can be a powerful tool, though many details remain to be validated and understood as this technique becomes more widely used.

###### Methods for ZooMS

Identification of species using collagen fingerprinting (i.e., ZooMS) can be paired with stable isotope analysis as both can typically be completed using collagen from the same bone sample. As with isotope analysis, ZooMS requires isolation and purification of the individual peptides within the collagen that are ultimately used to identify species (Buckley et al. 2014). Tandem MS is then utilized on the resulting peptide solution, with the relative timing and intensity (m/z) of the peaks in the mass spectrometry output (i.e., the abundance of the individual peptides) acting as a unique identifier or fingerprint for a species. This approach was developed, in part, for marine mammals and has been an effective tool for discerning species (Buckley et al. 2014).

The conventional approach for collagen extraction as described above is destructive and recently researchers have developed minimally invasive methods for ZooMS. The most effective method includes extracting collagen from bone powder collected from a film that polishes the surface of the bone (Evans et al. 2023). This bone powder then proceeds through a collagen extraction process and the rest of the ZooMS analysis as described above. These minimally invasive methods have been successfully used on whale bone identification (McGrath et al. 2026) and shows promise in reducing the destructions of specimens (see section *Special Considerations for Museum Collection Specimens* for further information).

#### Contaminants

##### Trace elements and heavy metals

Bone incorporates and stores trace elements differently than most other body tissues due to its mineral component. Both nonessential (including heavy metals) and essential elements are incorporated into the hydroxyapatite in the mineral matrix of the bone, but also in the hypermineralized cement lines that surround individual osteons (Beattie and Avenell 1992; Pemmer et al. 2013). Because many elements are readily incorporated into the crystalline lattice that makes up the mineral component of bone (Sharma et al. 2021), the skeleton may accumulate or house large fractions of the body’s total burden of some trace elements such as Pb, Sr, Ni, Zn, and Fe (Barry 1975; Yamamoto et al. 1987; Watanabe et al. 1996; Vighi et al. 2017). Bone can therefore be an important tissue to examine trace element concentrations; however, results should be interpreted with a thorough understanding of each element’s propensity to be incorporated into the bone matrix and the likely relationship among concentrations in bones and other tissues across taxa (Yamamoto et al. 1987; Watanabe et al. 1996; Correa et al. 2014; Vighi et al. 2017; Avery et al. 2023; Keenan et al. 2024). In addition, some elements can flux into and out of bone in soils and water (Misarti et al. 2011), therefore, the provenience and age of bones should be taken into consideration. Trace element analysis can be used to address research questions related to anthropogenic pollutants and natural sources of heavy metals, and can also provide insight into population structure, migration, health, and physiology.

Trace element concentrations have been measured in bones of numerous marine mammal species, including pinnipeds (Yamamoto et al. 1987; Watanabe et al. 1996; Saeki et al. 1999; Simokon and Trukhin 2021; Avery et al. 2023; Keenan et al. 2024), cetaceans (Cáceres-Saez et al. 2016; Vighi et al. 2017; Hao et al. 2020), and sirenians (Núñez-Nogueira et al. 2019). Though investigations into trace element concentrations in bones have largely related to biomonitoring and impact of anthropogenic input of certain elements into the environment (Vighi et al. 2017; Núñez-Nogueira et al. 2019; Borrell et al. 2023), trace element concentrations in bone may also reflect physiological processes and animal health (Honda et al. 1986; Hao et al. 2020).

###### Methods for trace elements and heavy metals

Methods to measure trace elements in tissues, including bone, have evolved over the past 60 years (Caroli et al. 1994). The “gold standard” for acquiring accurate concentrations of multiple elements in bone is inductively coupled plasma mass spectrometry (ICP-MS), which requires destructive methods to detect the low element concentrations in bone (Bolann et al. 2007). We suggest using approximately 100–220 mg of bone powder submitted for ICP-MS analysis to obtain a suite of bulk trace element data (Vighi et al. 2017; Hao et al. 2020; Simokon and Trukhin 2021). Typically, an acid digestion is required to breakdown the mineralized tissue to free the ions from the matrix and remove the organic material, which helps detection of elemental signals (Bolann et al. 2007). However, X-ray fluorescence (XRF) is a nondestructive method that uses X-rays to assess elements in a sample and cuts down on processing time and resources (Adesina et al. 2025a). ICP-MS and XRF trace element concentrations in bone strongly correlate suggesting XRF as another viable option (Adesina et al. 2025b).

##### Human contaminants (POPs and Microplastics)

Analysis of anthropogenically sourced contaminants in bone can be used to address research questions related to sources of contamination and how contaminants move through marine food webs, as well as the timing, location, and severity of human impacts on marine ecosystems.

Bones can serve as a tool to monitor persistent organic pollutant (POP) changes in marine mammals and their ecosystems. Numerous studies have assessed POPs and marine mammals (Khairy et al. 2021; Moorhead 2022), because they are a significant threat to population health (Stuart-Smith and Jepson 2017); however, baseline data on POPs in marine mammals prior to their ubiquitous use in the 1960s are still limited (Winfield et al. 2020). While certain POPs are preferentially deposited into lipid-rich tissues like blubber (Schantz et al. 1993; Tansel 2026), they are quantifiable in bone (Tanabe et al. 1981; Koskela et al. 2017) and may provide baseline data on POPs in marine mammal populations around the world.

Microplastics (MPs), defined as 1 µm–5.0 mm in size, are derived from the breakdown of human products (e.g., clothing, cosmetics) (Gregory 1996). They have been documented in marine ecosystems as potential pollutants to wildlife since the1970s (Carpenter and Smith 1972). Subsequent research has identified MPs as a particular concern due to their ability to disrupt endocrine function (Rochman et al. 2014), impact musculoskeletal and reproductive health (Fusagawa et al. 2024; Doroftei et al. 2025) and accumulate other pollutants for trophic transfer (Germanov et al. 2018; Prajapati et al. 2022). MP research is an emerging field, and they have been assessed in soft tissues of marine mammals; mostly scat, muscle, feces, gastrointestinal tract, and fatty tissues (i.e., blubber, melon) (Zantis et al. 2021; Merrill et al. 2023; Sletten et al. 2025; Vainberg and Abakumov 2025; Blade and Horstmann 2026).

Recently, MPs were discovered in human and rat skeletal bone, cartilage, and intervertebral discs (Yang et al. 2025). MPs are hypothesized to enter bone via blood and the circulatory system (Yang et al. 2025). Preliminary analyses show polar bear bone had the highest MP burden compared to other tissues (O’Connor et al. 2026) potentially making bone a valuable matrix to monitor plastic pollution in past and present marine mammal populations.

###### Methods for human contaminants (POPs and Microplastics)

The primary POPs measured in bones are perfluoroalkyl substances (PFASs) (Pérez et al. 2013; Koskela et al. 2016, 2017) with some research on other POPs (Tanabe et al. 1981). Studies have used around 1.0 g of pulverized bone, which is then either digested in sodium hydroxide (Pérez et al. 2013) or extracted using methanol and ammonium acetate (Koskela et al. 2016, 2017). The addition of individual POP internal standards is required during either method. Sample extracts need to go through chromatography (LC or GC) tandem mass spectrometry analysis (GC or LC/MS/MS) or other analytical methods (Vaye et al. 2022), because a suite of POPs of varying molecular complexity in a complex matrix (bone) is targeted during one analytical run.

MPs have only been measured in bones of humans and rats (Yang et al. 2025), and methods have recently been developed for polar bear bone (O’Connor et al. 2026). The digestion process consumes a minimum of 1.0–2.0 g of pulverized bone. Smaller sample weights will affect accuracy of MP quantification. Great care must be taken to avoid ubiquitous plastic contamination in any environment. Bone is digested in Fenton’s Reagent, a solution containing iron sulfate and hydrogen peroxide. Digested bone is then vacuum filtered onto glass fiber filters. Polymer identification of isolated MP particles can be accomplished using a variety of tools, such as Raman Micro-spectroscopy, Fourier Transform Infrared Spectroscopy, and pyrolysis (Santos et al. 2023; Yang et al. 2025; Blade and Horstmann 2026).

#### Genetics

##### Genetics

Bone provides an important source of DNA for marine mammal studies, especially when working with historical specimens, such as museum collections, or subfossil remains. DNA in bone binds to hydroxyapatite crystals within the mineral phase of bone organized with collagen fibrils (Campos et al. 2012; Kistler et al. 2017; James et al. 2020) (Fig. 1), where it remains until released during extraction or degraded by environmental processes. Bone preserves mitochondrial DNA sufficiently for sequencing even when degraded, enabling complete mitogenome reconstruction; however, DNA recovered from bone is often fragmented and chemically damaged, with a heightened risk of contamination from microbial, environmental, or human sources. Recovering nuclear DNA poses a particular challenge, because it occurs at lower copy numbers and degrades more quickly, leading to frequent genotyping failures at microsatellite loci (Wandeler et al. 2007). Success depends critically on preservation quality, and marine contexts present challenges, because saltwater exposure and dynamic oceanographic conditions accelerate DNA degradation compared to terrestrial burial environments (Allentoft et al. 2012). Genetic analysis of DNA from bone can be used to address research questions related to current and historical genetic diversity, bottleneck events, and population structure.

Multiple categories of biological information can be extracted from genetic material in marine mammal skeletons. Mitochondrial genetic variation is the most reliably recoverable data type, with mitochondrial haplotypes, haplotype diversity, nucleotide diversity, and phylogeographic structure routinely quantified from bone samples (Rosenbaum et al. 1997; Rosel et al. 2017). Temporal genetic diversity comparisons between historical and contemporary populations quantify changes in genetic variation attributed to exploitation, environmental shifts, and demographic events, such as prewhaling and postwhaling (Jackson et al. 2008). Nuclear genetic markers from bone, when recoverable, provide information about biparental inheritance patterns (Roze et al. 2005). Microsatellite genotypes can be used to assess heterozygosity, effective population size, and kinship, whereas genome-wide single nucleotide polymorphism (SNP) data allow finer-scale analysis of population structure and the reconstruction of demographic history (Foote et al. 2019).

###### Methods for DNA/RNA extraction

We recommend collecting approximately 100–250 mg, or more if wanting to perform multiple genetic analyses, of bone powder from dense cortical bone (Rohland and Hofreiter 2007). DNA extractions from marine mammal bone require disruption of the mineralized bone matrix to release preserved DNA molecules, while simultaneously removing inhibitory compounds that accumulate over time (Rohland and Hofreiter 2007). The most widely used method for ancient and historical bone samples employs EDTA-based decalcification followed by proteinase digestion and DNA purification steps (Rohland and Hofreiter 2007; Rohland et al. 2010). Alternatively, commercial DNA extraction kits designed for bone (e.g., Qiagen DNeasy protocols) perform as well or better for bone of varying quality (Rohland and Hofreiter 2007).

Bone DNA extraction requires several critical considerations. (1) The total DNA yield from ancient bone is typically very low (often < 1 µg) with highly fragmented molecules (<100 base pairs); (2) endogenous DNA is often < 5% of total DNA, with the remainder being environmental microbial contamination; and (3) inhibitory compounds co-extracted with DNA must be removed to enable downstream polymerase chain reaction (PCR) or sequencing reactions (Rohland and Hofreiter 2007). DNA concentration can be assessed via spectrophotometry or fluorometry, though these methods do not distinguish endogenous from contaminated DNA.

##### “Omics”

Functional genomic approaches beyond DNA sequence variation, including transcriptomics, proteomics, and epigenomics, remain difficult for marine mammal bone tissue due to the rapid degradation of RNA, metabolites, and epigenetic marks postmortem. RNA degrades within hours to days after death (Ferreira et al. 2018), precluding transcriptomic analysis from historical or archaeological bone that could otherwise reveal gene expression patterns related to physiology, stress response, or developmental stage. However, ancient protein analysis (paleoproteomics) has emerged as a viable alternative for identifying species and sex when DNA is too degraded for genetic analysis (see also ZooMS section) (Buckley et al. 2009; Welker et al. 2015; Rüther et al. 2022).

Epigenetic data, particularly DNA methylation patterns, represent an emerging frontier in ancient bone research, though applications to marine mammals remain limited. DNA methylation, the addition of methyl groups to cytosine bases, regulates gene expression and can address research questions related to developmental stage, environmental exposures, and physiological stress (Hernando-Herraez et al. 2013). Methylation-based epigenetic clocks have been successfully developed for age estimation in human bone, with studies showing strong correlations (*r* > 0.7) between methylation levels at cytosine–phosphate–guanine (CpG) sites and chronological age (Lee et al. 2020; Chen et al. 2025). Preliminary evidence from human studies suggest that methylation levels remain consistent across different skeletal elements within the same individual indicating potential applicability of bone-specific methylation models across various skeletal regions.

While methylation marks degrade over time through deamination processes that convert methylated cytosines to thymines, recent methodological advances have enabled partial recovery of ancient methylation patterns from well-preserved terrestrial mammal remains (Gokhman et al. 2014; Pedersen et al. 2014). For marine mammals, methylation analysis from bone faces additional challenges beyond those encountered with terrestrial specimens: saltwater exposure and marine taphonomic processes accelerate cytosine deamination. Despite these limitations, future applications of methylation analysis to exceptionally well-preserved marine mammal specimens could potentially reveal age-related methylation changes that serve as molecular aging markers (epigenetic clocks), providing life history information when traditional aging methods (e.g., tooth growth layer groups) are unavailable (Polanowski et al. 2014). The successful development of postmortem bone methylation clocks in humans (Chen et al. 2025) demonstrates proof-of-concept for skeletal epigenetics age estimation, though adaptation to marine mammals would require species-specific calibration and validation against known-age specimens.

###### Methods for “Omics”

DNA methylation analysis, such as epigenetics, from bone requires the same initial extraction as genetic studies (Section *Methods for DNA/RNA Extraction*), but with additional critical considerations. Methylation patterns must be captured before or during DNA extraction using bisulfite conversion, which chemically converts unmethylated cytosines to uracils, while leaving methylated cytosines (5-methycytosine) unchanged (Chen et al. 2025). Commercial kits (e.g., Qiagen EpiTect Fast DNA Bisulfite Kit) have been developed to perform bisulfite conversions for fresh or recently deceased specimens. The bisulfite-converted DNA is then amplified using methylated-specific PCR primers targeting species- and question-specific CpG sites in genes, followed by pyrosequencing to quantify methylation levels at specific sites (Chen et al. 2025). The proteinase digestion step used in standard bone DNA extraction also releases degraded collagen peptides and other proteins, allowing paleoproteomic samples to be recovered alongside DNA from the same extraction (Rusu et al. 2019). Unlike ZooMS, which is largely limited to species identification via collagen peptide mass fingerprinting, broader shotgun proteomic approaches are being extended to include sex identification (Rüther et al. 2022).

Distinguishing between true ancient methylation signals from postmortem cytosine deamination damage presents a major analytical challenge for ancient or degraded bone. Deamination of cytosine to uracil occurs naturally during DNA degradation and is indistinguishable from bisulfite-induced conversion, creating ambiguity about whether observed patterns represent genuine ancient methylation or taphonomic artifacts (Pedersen et al. 2014). Marine samples face additional challenges: saltwater exposure accelerates cytosine deamination rates compared to terrestrial burial environments, further complicating methylation inferences from marine mammal bone. Computational approaches to distinguish ancient methylation from damage require very high-coverage sequencing data (typically > 30 × genome coverage) that is rarely achievable with degraded marine mammal bone DNA (Gokhman et al. 2014).

Due to these substantial technical limitations, DNA methylation analysis from marine mammal bone is currently recommended only for: (1) fresh or frozen tissue samples collected within days of death, where postmortem deamination is minimal; (2) exceptionally well-preserved specimens from permafrost or other optimal taphonomic contexts; (3) pilot studies explicitly designed to assess methylation signal preservation in marine bone under different environmental conditions. Researchers interested in developing methylation-based age estimation (epigenetic clocks) for marine mammal bone should consult established protocols from human forensic bone studies (Lee et al. 2020; Chen et al. 2025) but must conduct species-specific validation with known age specimens and assess whether marine burial conditions permit reliable methylation signal recovery.

## Bone studies

Archaeologists (study of past human life and cultures), palaeoecologists (study of ancient organisms, ecosystems, and environments), and palaeontologists (study of ancient nonhuman life using fossilized remains), have long studied bone as one of the few mediums beyond soils and sediments that survive for thousands of years. Archaeologists in particular have focused on bone remains in sites to quantify species present, minimum number of identified specimens of each species, minimum number of individuals, size of an individual, meat weight for prey, etc. (Grayson 1984). These can all be accomplished without destructive analyses and yield information for biological, ecological, and archeological studies. In the last few decades, archaeologists and physical anthropologists have adopted many of the instrumental analyses used in medical and ecological fields, pioneered on muscle, blood, plant, and other tissues, specifically to study bones (Ambrose and Krigbaum 2003; Lee-Thorp 2008; Pollard et al. 2020; Simpson et al. 2021), with an emphasis on stable isotopes and their value to reconstructing diets, climate, and ecosystems (Stevens et al. 2025). While covering the different aspects of these disciplines (e.g., palaeoecologists vs. palaeontologists) is outside the scope of this paper, it should be acknowledged these fields provide the backbone of methods used in marine mammal bone studies.

### Where do you find bone specimens for studies?

#### Museum collections

Natural history museum archives collect biological specimens, which are instrumental in conservation research. Though work has been conducted for many years (Alberch 1993), these collections are becoming even more important as human disturbances to natural ecosystems and the need to assess past populations increase (Suarez and Tsutsui 2004; Ferguson 2020; Salvador and Cunha 2020; Norris and Tweddle 2026). Mammal collections have grown and evolved through time resulting in a wealth of specimens available for research (Cook and Light 2019). For example, the largest mammal collection in the Western Hemisphere is the United States National Museum of Natural History with ∼600,000 specimens. These massive collections have online databases that can be searched by species and tissue type; however, direct communication with curators is crucial to obtain the most current specimen information (Cook and Light 2019), as well as access to additional rare marine mammal specimens to increase sample sizes (Smith et al. 2021a). A summary of museums with mammal collections, estimates for number of specimens in each collection, and links to access digital museum resources can be found in Cook and Light (2019).

There are available papers to familiarize researchers with destructive sampling requests of historical bone (Freedman and Dorp 2018); however, museums typically have their most current application processes online (e.g., National Museum of Natural History 2026). This approval process can take significant time, so it is highly encouraged to submit your application long before any hard deadlines. Once approved, a museum will either require that qualified researchers (typically approved by museums) travel to the institution to sample their approved specimens, or in rare cases, they will allow a loan of materials shipped to researchers or their institutions. Museums will also require the return of any unused bone, or bone material, such as collagen, within a certain time period after project completion (Freedman and Dorp 2018).

##### Special considerations for museum collection specimens

Special considerations are required for sampling archaeological bones. We recommend collaborating with an archaeologist if accessing archaeological samples, as this expertize is essential to understand provenience, context, material dating, and the accurate identification of individuals and species within a collection. Archaeological methods for excavation and curation are well established (Grayson 1984), and sampling without a clear understanding of context can significantly compromise data interpretation. Archaeologists/anthropologists or museum curators also have knowledge of agreements and permissions set in place with descendant communities; sampling without consideration of any such agreements is unethical.

Destructive sampling of zooarchaeological material (animal remains) is on the rise due to advancement in methods, such as those discussed in this manuscript. This rise in destructive sampling poses significant ethical considerations since bones are limited resources. We suggest following guidelines that help minimize negative effects and consider the ethics of destructive sampling in zooarchaeological material, such as marine mammal bones. This includes the following steps summarized from Pálsdóttir et al. (2019):

1. **Collaborate**: As stated previously, it is important to collaborate with museum curators, archaeologists, and any other expert that can help identify usable specimens and ensure the most value is obtained through any destructive sampling that occurs.
2. **Have a sampling strategy**: Know the amount of bone needed for your targeted analyses. We give recommended amounts for each analysis discussed in this manuscript and how some of these analyses may be paired for greater efficiency and reduced impact to specimens. Sample masses stay within a commonly used range of 1.0–1.5 g.
3. **Know your samples**: Be familiar with how your specimens were stored and preserved, as well as their estimated date and time of collection/death. We discuss this further in the “Sampling considerations for marine mammal bone” section.
4. **Permissions**: Obtain the necessary permissions from all stakeholders. This includes museums, collaborators, Indigenous communities (if applicable), and any other stakeholder. Be aware of the destructive sampling policies for each institution.
5. **Documentation**: Take relevant photos, measurements, and high-quality photographs of the specimens *before* and *after* you destructively sample a specimen.
6. **Sample size:** Sample only what is required for targeted analyses. Again, we provide a blueprint for destructive analyses with a range of 1.0–1.5 g.
7. **Traceability**: Make sure raw data is traceable back to specimens in the respective institutions. This typically requires specimen IDs included along with raw or processed data in manuscript(s). This avoids repeat sampling and analyses of specimens.
8. **Know what is in your laboratory**: As samples are collected and brought to your lab, register each sample in a database and set up an organized storage system for samples. Note what samples contain leftover material that may have to be returned to its respective institution.
9. **Share generated data**: Publish data open access and provide the natural history museum or collection institution copies of all publications that include their specimens’ data.
10. **Publish negative results**: Negative results are critical to inform other researchers of what has been attempted and what does not work. This again helps avoid repeat sampling/destruction of specimens.

This is a summary of considerations for scientists that intend on incorporating zooarchaeological material, such as bones, into their research portfolio. We recommend consulting Pálsdóttir et al. (2019) as you develop your plan for sampling marine mammal bones from various Natural History institutions.

#### Stranded animals

Lethal sampling, or the killing of marine mammals solely for scientific research is largely unpopular, unethical, and in most cases illegal, in the research community (Gales et al. 2009). Alternative methods are used to acquire samples that cannot be collected from live animals, such as bone, including collaborations with Indigenous communities (see below), as well as sampling fisheries bycatch and stranding events (Gales et al. 2009).

Marine mammal stranding networks around the world respond to beach cast animals generating a wealth of knowledge about marine mammals (Gulland et al. 2025; Petitguyot et al. 2025). While development of robust stranding programs varies globally, generally, federal governments or regional networks support local stranding partners that carry out day-to-day operations. These local networks coordinate sample collection and can transfer materials (e.g., bone) to researchers as per their stranding agreements with their regional or governmental partners (Gulland et al. 2025; Petitguyot et al. 2025). The best first step for obtaining samples from stranding networks is to reach out to a local network and inquire about sample requests.

#### Indigenous community collaborations

Indigenous communities have an intricate knowledge of marine mammal biology and ecology due to their rich cultures and histories with marine mammal subsistence around the world (Marsh et al. 2022; Metcalf 2024). For example, Alaska Native communities co-regulate certain marine mammals with the federal and state government. The Eskimo Walrus Commission (EWC) is an example of a recognized statewide organization composed of members from coastal communities dependent on Pacific walruses for subsistence that collaborates with U.S. Fish and Wildlife Service (USFWS) officials to co-manage walrus population health and subsistence harvest (Metcalf et al. 2025). By contacting tribal leaders or tribal commissions (e.g., EWC) with project ideas that support their efforts in marine mammal conservation, it is possible to collaborate with Indigenous communities to obtain samples, such as bones (Charapata et al. 2018, 2021; Clark et al. 2019a, 2019b; Mills et al. 2024). Tribal permissions are required, and it is highly recommended to contact tribes directly through appropriate channels (e.g., Bureau of Indian Affairs 2026) prior to beginning any new research project anticipating community collaboration.

There are many more examples of western scientists collaborating with Indigenous communities to study marine mammals including from New Zealand (Elmahdy et al. 2025), Canada (Breton-Honeyman et al. 2021), and South America (Pozzi and Ladio 2023). This section largely focuses on Indigenous collaborations with North American communities, specifically Native Alaskan communities, due to the authors’ areas of experience; however, ethical collaboration with Indigenous communities is a critical consideration in many areas of the world and time should be spent to develop an understanding local histories, dynamics, and relationships that may apply to your intended research before beginning work.

### Limitations of bone studies

There are limitations to any research approach, and the restrictions associated with studies of bone samples should be acknowledged. The primary consideration is bone quality; defined here as postmortem preservation of a bone’s composition (Kendall et al. 2018), as poor quality may limit certain analyses. For example, collagen and lipids are robustly preserved in freshly sampled or properly archived bones compared to archaeological bones that have been exposed to acids and other soil contaminants, which may result in poor quality for stable isotopes (Guiry and Szpak 2020) and hormones (Charapata et al. 2018). While bones can be collected from different sources, most samples are archived in museums. When permission is granted to destructively sample museum bones, the amount of bone is limited to only enough to complete the requested analyses, typically 1.0–1.5 g. Careful planning and coordination can result in efficient data acquisition from these small sample masses (see section *Researcher’s Guide to Addressing Their Questions*).

It is unknown how most biomarkers or parameters of interest vary across skeletal elements, but variability is likely high. Some studies show stable isotopes and hormones are similar across some elements (stable isotopes: Riofrío-Lazo and Aurioles-Gamboa 2013; Clark et al. 2017; hormones: Yarrow et al. 2010; Charapata et al. 2018); however, given differences in turnover rates among skeletal elements (Allen and Burr 2019; Quinn et al. 2025), this cannot be assumed for all parameters of interest or all skeletal elements (Keenan et al. 2024; Schrader et al. 2026). That said, limited sample availability means assuming a single bone sample is representative of an animal is often a necessary assumption but should be acknowledged as a caveat (Charapata et al. 2021). Studies show that sampling different bones in one consistent location (e.g., always sampling on the ventral left side of mandibles) helps control some variability (Smith et al. 2021b); however, sometimes only bone fragments are available to sample. Thus, consistency of sampling to reduce variability must be balanced with appropriate sample size concerns and any such decisions should be acknowledged.

Bones, especially those from archaeological collections, typically do not come with extensive metadata (sex, age class, species); however, there are ways to assess this within the design of the study, including assessing fusion of bones for age class and morphological measurements for sex and species (Fig. 3) (Grayson 1984; Taylor et al. 2020; Smith et al. 2021a; Borrell et al. 2023; Dierickx et al. 2025). Bones slated for inclusion in studies should at least have a known location and an associated date with additional metadata being obtained within the scope of the broader study.

### Sampling considerations for marine mammal bone

It is important to expect a high probability of success when requesting to destructively sample bone archived in collections due to the limited and irreplaceable nature of these resources. Bone quality can be maintained by storing specimens in a cool and dry place relatively soon after collection. No boiling or chemical treatments should be applied and, if excavated from an archaeological site, the degree of degradation should be closely assessed by an expert. Additionally, proper cleaning procedures of soft tissues (Cáceres-Saez et al. 2016) and surface contaminants (Charapata et al. 2018) help to preserve biological data in the bone. Most museums’ destructive sampling requests only allow a small amount of bone to be sampled, so one should assume 1.0–1.5 g of bone may be available for analysis. Sterile conditions are required for certain analyses (e.g., genetics, microplastics), and we suggest consulting analyte-specific sampling methods using marine mammal tissues (Fulton 2012; Stock et al. 2019). It is recommended bones used in research have basic associated metadata including collection location and year, sex, and age class. These sampling considerations result in a “best case” museum-based scenario, when sampling a bone for biological data. Archaeological bones are the most difficult to assess in terms of quality due to unknown burial periods, environmental conditions, and other parameters that need to be considered (e.g., sex, age class) (Grayson 1984).

For marine mammal studies in particular, the origin and death of the study animal is critical for data interpretation. For example, obtaining samples from Indigenous subsistence harvests ensures that the individual was part of the healthy, free-ranging population, although hunter preferences and biases when selecting animals have to be considered. On the other hand, samples collected from dead stranded individuals may not be representative of the general population.

### Questions addressed with an integrated bone dataset

#### Case study: how will walruses respond to a rapidly changing arctic ecosystem?

Archaeological, historical, and modern bone was used in an extensive Pacific walrus research project that aimed to understand the adaptability of walruses to a rapidly changing Arctic using a multi-disciplinary approach. Bones from museums, such as the University of Alaska Museum and Smithsonian Institution National Museum of Natural History provided bones from archaeological (as old as ∼4000 calendar years before present) up to historical (2000s CE) time periods, while collaborating Indigenous communities donated contemporary (2013–2016 CE) bone samples.

Multiple researchers across various disciplines efficiently worked together to sample individual walrus bones. One sampling event per bone resulted in a range of data for each individual including stable isotopes (δ^15^N and δ^13^C), mtDNA, hormones, and morphological measurements for sex and age class identification (Fig. 3). The morphometrics and sex analyses made metadata more robust when analyzing the stable isotopes, mtDNA, and hormone datasets. The following primary research questions were addressed using walrus bone and the integrated datasets presented in Fig. 3.

**Method Development**

1) Can morphometrics of Pacific walrus skulls and mandibles help identify sex of unknown specimens? (Fig. 3A)

**Main Finding**: Yes, collecting measurements of skulls and mandibles from known males and females allowed researchers to assign sex to other walrus skulls and mandibles with unknown sex data, including archaeological specimens. This did not require destructive analysis (Taylor et al. 2020).

2) Are stable isotopes (δ^15^N and δ^13^C) different among paired walrus bones? (Fig. 3B)

**Main Finding**: No, δ^15^N and δ^13^C were similar among paired cranium and mandible samples allowing for direct comparison of isotope data between these two commonly used bone elements in marine mammal research (Clark et al. 2017).

**Ecology**

1) Have walruses significantly changed their diet in response to low sea ice conditions? (Fig. 3C)

**Main Finding**: Evidence from δ^15^N and δ^13^C, indicators of foraging trophic level and location, respectively, suggest that Pacific walruses did not substantially alter their diets between previous periods of high and low sea ice. This was interpreted as an indicator of resiliency to environmental change (Clark et al. 2019a).

**Physiology**

1) Does reduced sea ice result in changes to reproductive and stress physiology? (Fig. 3D)

**Main Finding**: Yes, reduced sea ice conditions correlated with lower progesterone in female walrus bones suggesting lower reproductive activity during periods with less sea ice. However, cortisol (biomarker of stress) did not show any strong correlations with sea ice indicating no strong stress response to low sea ice conditions (Charapata et al. 2021).

**Genetic**

**Fig. 4:**
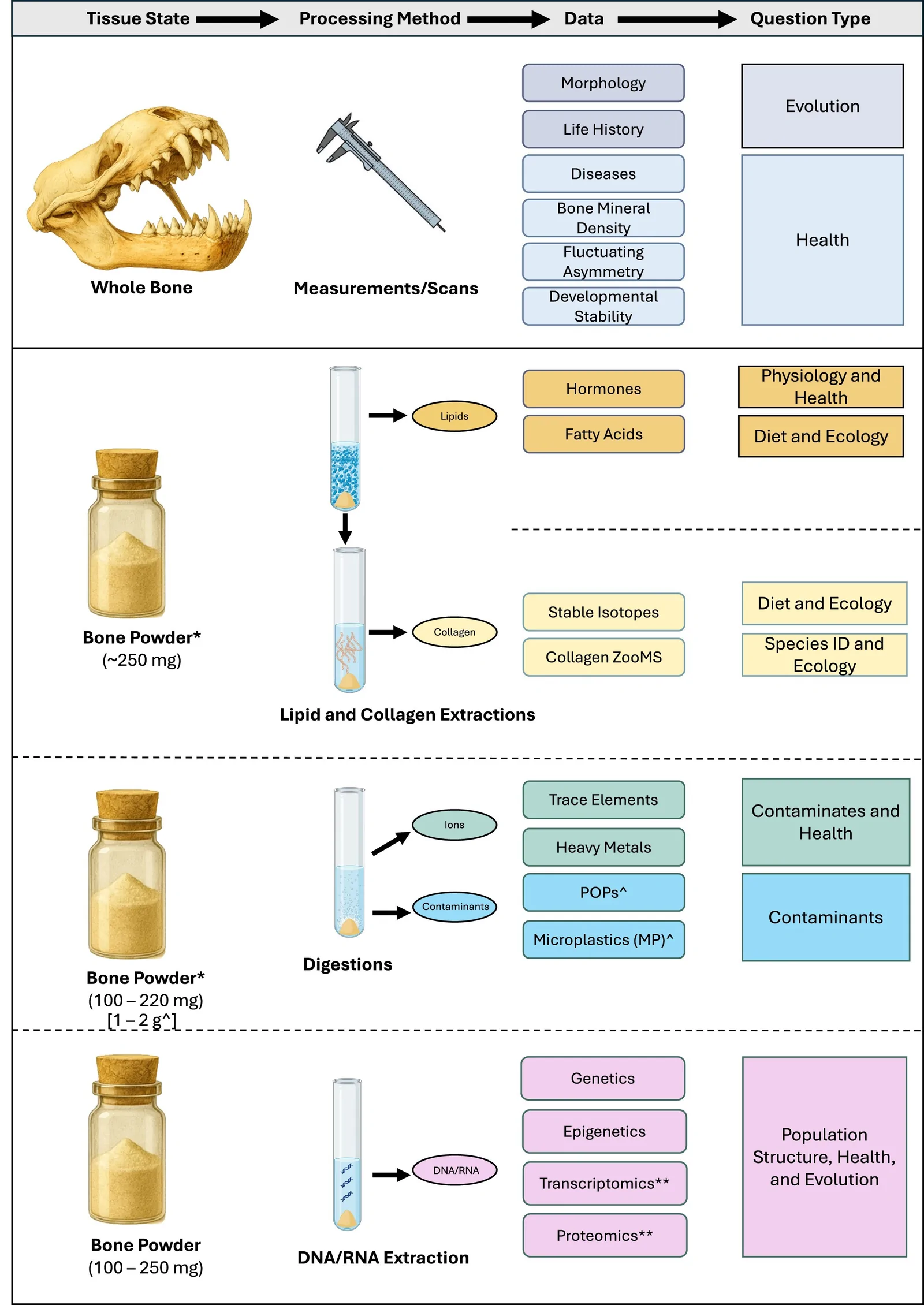
Flowchart of different analytical approaches that can be performed on marine mammal bone using relatively small (∼1.5 g) sample mass. * Indicates powder or solid bones are usable in processing methods. **Transcriptomic data are recoverable only from fresh or recently deceased tissue; postmortem RNA degradation precludes this analysis in historical or archaeological bone and proteomics here refers to shotgun/LC-MS/MS-based approaches recovered alongside DNA extraction, distinct from the collagen peptide mass fingerprinting (ZooMS) shown above. The images depicting “bone powder” and “calipers” were generated using artificial intelligence (e.g., Microsoft Copilot).

1) If the walrus population does decline due to lack of sea ice, will this cause a genetic bottleneck? (Fig. 3E) (Fig. 4).

**Main Finding**: No, genetic diversity was not significantly reduced during periods of past stress and population declines, specifically commercial hunting of walruses that greatly reduced population size. This example of genetic resiliency signifies genetic diversity would most likely remain high during periods of low sea ice and/or population declines (Mills et al. 2024).

Overall, these results suggested that walruses are highly adaptable to environmental change. Yet, continued monitoring is warranted since future warming scenarios presents risks to walrus population health (Johnson et al. 2024).

#### Researcher’s guide to addressing their questions

Researchers can address a wide range of questions using the multi-disciplinary data obtainable from a relatively small sample mass (1.0–1.5 g) of bone as demonstrated with the walrus case study in the previous section. We provide a flowchart of estimated masses that can be used for various analyses, while accounting for different processing steps (Fig. 4). Some analyses, such as hormones and stable isotopes, do not even need their own portion of bone mass, because they can be obtained from the same ∼250 mg sample (Fig. 4). This flowchart serves as a general guide when developing a research project using bone; however, appropriate literature and subject matter experts should be consulted when deciding which specific methods to employ to appropriately addresses research questions. For example, ice-associated marine mammals like polar bears and ice seals would greatly benefit from a similar bone study like the walrus case study described above because they deal with similar stressors as a result of anthropogenic activity.

## Future research and improvements

While there have been major advances in conservation studies using bone, many areas will benefit greatly from additional research. Estimates of marine mammal bone turnover rates are rare and difficult to obtain but may be feasible for certain periods of life using data proxies, such as stable isotopes (Newsome et al. 2006). Future studies should focus on how bone turnover impacts physiological, ecological, contaminant, and genetic data measured in bones. For example, hormone and fatty acid storage periods in bone are most likely associated with lipid turnover rates, whereas stable isotope values are driven by collagen turnover. Thus, understanding the time signature the stable isotopes and hormones represent from a bone sample will help with interpretation of data (Fleming et al. 2018).

Standardizing methods to obtain a variety of data from bone will improve comparisons across studies. While analytical techniques will continue to develop, improve, and become more efficient, standardizing how, where, and which component of a bone is sampled will provide basic standards for processing bones slated for different analyses. For example, it is recommended that bone collagen is extracted, purified, and then analyzed for stable isotope analyses (Guiry and Szpak 2020; Teixeira et al. 2022). A systematic review of methods for each data type will be greatly beneficial.

Though not described in this manuscript, other mineralized tissues preserved for millennia, such as teeth, show immense promise in assessing retrospective data of various marine mammal species. Teeth of marine mammals typically accrue light and dark annual growth layer groups in their dentin and/or cementum layers that can be individually sampled to assess lifetime patterns of trace elements (Clark et al. 2020, 2021b), hormones (Hudson et al. 2021), stable isotopes (Teixeira et al. 2022), and possibly genetics (Pichler et al. 2001). Teeth share many characteristics with bone, and the detailed information about life history, ecology, physiology, and movement that can be accessed by examining individual tooth growth layer groups suggests that pairing these structures with analysis of bones may be a powerful tool. Further, the methods developed for tooth analysis may have direct application for bones or other structures that accrue growth layer groups, and application of these approaches (e.g., laser ablation of cross-sectioned bone growth layer groups) should be explored further.

## Conclusions

Bone persists for millennia, preserves ecological, physiological, contaminant, and genetic data, and is commonly archived in museums and other collections making it an ideal tissue to assess marine mammal conservation. Bone allows researchers to “go back in time” to assess various population baselines (e.g., ecological and physiological) prior to global human alterations to marine ecosystems. Additionally, acquiring a time-series of data allows for assessment of past populations’ resiliency to ecological stressors and provides insight into adaptability to climate change. This unique strategy for conservation research is becoming more valuable to researchers with the certainty of continued warming of the planet (IPCC 2022). While there is more to be done, we hope to provide researchers with an introduction on how to effectively incorporate bone into their marine mammal conservation studies.

## Data Availability

No new data were generated from this study.

## Notes

From the symposium “Hormones at sea: New frontiers in marine mammal endocrinology and ecophysiology” presented at the annual meeting of the Society for Integrative and Comparative Biology, January 3–7th, 2026.

## References

[bib1] Ackman RG, Eaton CA, Litchfield C. 1971. Composition of wax esters, triglycerides and diacyl glyceryl ethers in the jaw and blubber fats of the Amazon River dolphin (*Inia geoffrensis*). Lipids. 6:69–77. 10.1007/BF02531319. 5548640

[bib2] Adesina KE, Burgos CJ, Grier TR, Sayam ASM, Specht AJ. 2025a. Ways to measure metals: from ICP-MS to XRF. Curr Environ Health Rep. 12:7. 10.1007/s40572-025-00473-y. 39865194 PMC11913532

[bib3] Adesina KE, Parducci SA, Brain JD, Molina RM, Weisskopf M, Specht AJ. 2025b. Comparison between inductively coupled plasma-mass spectrometry and benchtop X-ray fluorescence performance for trace elemental exposure in rat tissues. J Trace Elem Miner. 12:100229. 10.1016/j.jtemin.2025.100229. PMC12164575 40520248

[bib4] Alberch P . 1993. Museums, collections, and biodiversity inventories. Trends Ecol Evol. 8:372–5. 10.1016/0169-5347(93)90222-B. 21236198

[bib5] Allen MR, Burr DB. 2019. Bone growth, modeling, and remodeling. In: Basic and applied bone biology. Amsterdam, Netherlands: Elsevier. p. 85–100.

[bib6] Allentoft ME, Collins M, Harker D, Haile J, Oskam CL, Hale ML, Campos PF, Samaniego JA, Gilbert TPM, Willerslev E et al. 2012. The half-life of DNA in bone: measuring decay kinetics in 158 dated fossils. Proc R Soc B Biol Sci. 279:4724–33. 10.1098/rspb.2012.1745. PMC3497090 23055061

[bib7] Ambrose SH, Krigbaum J. 2003. Bone chemistry and bioarchaeology. J Anthropol Archaeol. 22:193–9. 10.1016/S0278-4165(03)00033-3.

[bib8] Ames AL, Van Vleet ES, Reynolds JE. 2002. Comparison of lipids in selected tissues of the Florida manatee (Order Sirenia) and bottlenose dolphin (Order Cetacea; Suborder Odontoceti). Comp Biochem Physiol Part B. 132:625–34. 10.1016/S1096-4959(02)00082-9. 12091108

[bib9] Amson E, Bibi F. 2021. Differing effects of size and lifestyle on bone structure in mammals. BMC Biol. 19:1–18. 10.1186/s12915-021-01016-1. 33926429 PMC8086358

[bib10] Anderson S . 2004. Soxtec: its principals and applications. In: Luthria D, editor. Oil extraction and analysis: critical issues and comparative studies. Champaign, IL: AOCS Press. p.11–25. 10.1201/9781439822340.

[bib11] Atkinson S . 2020. Endocrine profiling of reproductive status and evidence of pseudopregnancy in the Pacific walrus (*Odobenus rosmarus divergens*). PLoS One. 15:1–19. 10.1371/journal.pone.0239218 PMC7491731 32931507

[bib12] Avery JP, Castellini JM, Misarti N, Keenan M, Gastaldi A, Funk C, O’Hara TM, Rea LD. 2023. Evaluating methods for determining mercury concentrations in ancient marine fish and mammal bones as an approach to assessing millennial-scale fluctuations in marine ecosystems. Front Ecol Evol. 11:1–19. 10.3389/fevo.2023.1251282.38516293

[bib13] Barratclough A, McFee WE, Stolen M, Hohn AA, Lovewell GN, Gomez FM, Smith CR, Garcia-Parraga D, Wells RS, Parry C et al. 2023. How to estimate age of old bottlenose dolphins (Tursiops truncatus); by tooth or pectoral flipper?. Front Mar Sci. 10. 10.3389/fmars.2023.1135521.

[bib14] Barry PSI . 1975. A comparison of concentrations of lead in human tissues. Br J Ind Med. 32:119–39. 1131339 10.1136/oem.32.2.119 PMC1008038

[bib15] Bas M, García NA, Crespo EA, Cardona L. 2020. Intraskeletal variability in stable isotope ratios of C and N among pinnipeds and cetaceans. Mar Mamm Sci. 36:375–85. 10.1111/mms.12644.

[bib16] Beattie JH, Avenell A. 1992. Trace element nutrition and bone metabolism. Nutr Res Rev. 5:167–88. 10.1079/NRR19920013. 19094319

[bib17] Biga LM, Bronson S, Dawson S, Harwell A, Hopkins R, Kaufmann J, LeMaster M, Matern P, Morrison-Graham K, Oja K et al. 2025. Bone tissue and the skeletal system. In: Biga LM, editor. Anatomy and physiology 2e. 2nd ed Corvalis, OR: Oregon State University.

[bib18] Black JD, Tadros BJ. 2020. Bone structure: from cortical to calcium. Orthop Trauma. 34:113–9. 10.1016/j.mporth.2020.03.002.

[bib19] Blade T, Horstmann L. 2026. Microplastics in muscle and blubber of the Pacific walrus (*Odobenus rosmarus divergens*). Mar Env Res. 215:107825. 10.1016/j.marenvres.2025.107825. 41478058

[bib20] Bolann BJ, Rahil-Khazen R, Henriksen H, Isrenn R, Ulvik RJ. 2007. Evaluation of methods for trace-element determination with emphasis on their usability in the clinical routine laboratory. Scand J Clin Lab Invest. 67:353–66. 10.1080/00365510601095281. 17558890

[bib21] Borrell A, Garcia-Garin O, Aguilar A, Vighi M, Valdivia M, González EM, Páez-Rosas D, Drago M. 2023. High aluminum content in bone of marine mammals and its relation with source levels and origin. Environ Pollut. 331:121936. 10.1016/j.envpol.2023.121936. 37263563

[bib22] Breton-Honeyman K, Huntington HP, Basterfield M, Campbell K, Dicker J, Gray T, Jakobsen AER, Jean-Gagnon F, Lee D, Laing R et al. 2021. Beluga whale stewardship and collaborative research practices among indigenous peoples in the Arctic. Polar Res. 40:5522. 10.33265/polar.v40.5522.

[bib23] Buckley M, Collins M, Thomas-Oaies J, Wilson JC. 2009. Species identification by analysis of bone collagen using matrix-assisted laser desorption/ionisation time-of-flight mass spectrometry. Rapid Commun Mass Spectrom. 23:3843–54. 10.1002/rcm.4316. 19899187

[bib24] Buckley M, Fraser S, Herman J, Melton ND, Mulville J, Pálsdóttir AH. 2014. Species identification of archaeological marine mammals using collagen fingerprinting. J Archaeol Sci. 41:631–41. 10.1016/j.jas.2013.08.021.

[bib25] Bureau of Indian Affairs . 2026; Bureau of Indian Affairs—Tribal Leaders Directory. https://biamaps.geoplatform.gov/Tribal-Leaders-Directory/ (16 February 2026, date last accessed).

[bib26] Cáceres-Saez I, Panebianco MV, Perez-Catán S, Dellabianca NA, Negri MF, Ayala CN, Goodall RNP, Cappozzo HL. 2016. Mineral and essential element measurements in dolphin bones using two analytical approaches. Chem Ecol. 32:638–52. 10.1080/02757540.2016.1177517.

[bib27] Campos PF, Craig OE, Turner-Walker G, Peacock E, Willerslev E, Gilbert MTP. 2012. DNA in ancient bone—Where is it located and how should we extract it?. Ann Anat. 194:7–16. 10.1016/j.aanat.2011.07.003. 21855309

[bib28] Cani A, Cardona L, Valdivia M, González EM, Drago M. 2023. Niche partitioning among marine mammals inhabiting a large estuary as revealed by Stable Isotopes of C, N, S, and O. Estuaries Coasts. 46:1083–97. 10.1007/s12237-023-01193-y.

[bib29] Capulli M, Paone R, Rucci N. 2014. Osteoblast and osteocyte: games without frontiers. Arch Biochem Biophys. 561:3–12. 10.1016/j.abb.2014.05.003. 24832390

[bib30] Caroli S, Alimonti E, Coni F, Petrucci O, Senofonte O, Violante N. 1994. The Assessment of Reference Values for Elements in Human Biological Tissues and Fluids: a Systematic Review. Crit Rev Anal Chem. 24:363–98. 10.1080/10408349408048824.

[bib31] Carpenter EJ, Smith KL. 1972. Plastics on the Sargasso sea surface. Science (1979). 175:1240–1. 10.1126/science.175.4027.1240 5061243

[bib32] Charapata P, Horstmann L, Jannasch A, Misarti N. 2018. A novel method to measure steroid hormone concentrations in walrus bone from archaeological, historical, and modern time periods using liquid chromatography/tandem mass spectrometry. Rapid Commun Mass Spectrom. 32:1999–2023. 10.1002/rcm.8272. 30192037 PMC6282614

[bib33] Charapata P, Horstmann L, Misarti N. 2021. Steroid hormones in Pacific walrus bones collected over three millennia indicate physiological responses to changes in estimated population size and the environment. Conserv Physiol. 9:1–20. 10.1093/conphys/coaa135. PMC7836870 33537147

[bib34] Charapata P, Oxman D, McNeel K, Keith A, Mansouri F, Trumble S. 2022. Lifetime hormone profiles for a long-lived teleost: opercula reveal novel estimates of age-specific reproductive parameters and stress trends in yelloweye rockfish (*Sebastes ruberrimus*). Can J Fish AquatSci. 79:1712–28. 10.1139/cjfas-2022-0048.

[bib35] Charapata P, Trumble S. 2023. Environmental and ecological changes influence lifetime trends of reproduction, stress, and stable isotopes reconstructed from female yelloweye rockfish opercula. ICES J Mar Sci. 80:1500–15. 10.1093/icesjms/fsad078.

[bib36] Chen S, Zhang C, Wen D, Wang C, Tang X, Li X, Fu X, Li J, Jin X, Luo H et al. 2025. Epigenetic age signatures in postmortem rib samples. Electrophoresis. 46:1397–406. 40799162 10.1002/elps.70014

[bib37] Clark CT, Finney BP, Cape MR, Misarti N, Shapley MD, Mueter FJ, Clark CT. 2021a. SuessR: regional corrections for the effects of anthropogenic CO_2_ on δ^13^C data from marine organisms. Methods Ecol Evol. 2021:1–13.

[bib38] Clark CT, Horstmann L, de Vernal A, Jensen AM, Misarti N. 2019a. Pacific walrus diet across 4000 years of changing sea ice conditions. Quat Res. 108:26–42. 10.1017/qua.2018.140.

[bib39] Clark CT, Horstmann L, Misarti N. 2017. Quantifying variability in stable carbon and nitrogen isotope ratios within the skeletons of marine mammals of the suborder Caniformia. J Archaeol Sci Rep. 15:393–400.

[bib40] Clark CT, Horstmann L, Misarti N. 2019b. Lipid normalization and stable isotope discrimination in Pacific walrus tissues. Sci Rep. 9:5843. 10.1038/s41598-019-42095-z. 30971722 PMC6458160

[bib41] Clark CT, Horstmann L, Misarti N. 2020. Zinc concentrations in teeth of female walruses reflect the onset of reproductive maturity. Conserv Physiol. 8:1–13. 10.1093/conphys/coaa029. PMC7154182 32308984

[bib42] Clark CT, Horstmann L, Misarti N. 2021b. Walrus teeth as biomonitors of trace elements in Arctic marine ecosystems. Sci Total Environ. 772:145500. 10.1016/j.scitotenv.2021.145500. 33571762

[bib43] Clarke B . 2008. Normal bone anatomy and physiology. Clin J Am Soc Nephrol. 3:131–9. 10.2215/CJN.04151206. PMC3152283 18988698

[bib44] Colldén H, Nilsson ME, Norlén A-K, Landin A, Windahl SH, Wu J, Gustafsson KL, Poutanen M, Ryberg H, Vandenput L et al. 2022. Comprehensive sex steroid profiling in multiple tissues reveals novel insights in sex steroid distribution in male mice. Endocrinology. 163:bqac001. 10.1210/endocr/bqac001. 34999782 PMC8807178

[bib45] Colonese AC, Farrell T, Lucquin A, Firth D, Charlton S, Robson HK, Alexander M, Craig OE 2015. Archaeological bone lipids as palaeodietary markers. Rapid Commun Mass Spectrom. 29:611–8. 10.1002/rcm.7144. 26212278

[bib46] Colonese AC, Lucquin A, Guedes EP, Thomas R, Best J, Fothergill BT, Sykes N, Foster A, Miller H, Poole K et al. 2017. The identification of poultry processing in archaeological ceramic vessels using in-situ isotope references for organic residue analysis. J Archaeol Sci. 78:179–92. 10.1016/j.jas.2016.12.006.

[bib47] Consoli FMA, Reyes-Matute A, Rivero MA, Encinoso M, Fernandez A. 2025. Osteonecrosis in pilot whales (*Globicephala macrorhynchus*). Res Vet Sci. 192:105705. 10.1016/j.rvsc.2025.105705. 40412346

[bib48] Cook JA, Light JE. 2019. The emerging role of mammal collections in 21st century mammalogy. J Mammal. 100:733–50. 10.1093/jmammal/gyy148.

[bib49] Coombs EJ, Clavel J, Park T, Churchill M, Goswami A. 2020. Wonky whales: the evolution of cranial asymmetry in cetaceans. BMC Biol. 18:86. 10.1186/s12915-020-00805-4. 32646447 PMC7350770

[bib50] Correa L, Rea LD, Bentzen R, O’Hara TM. 2014. Assessment of mercury and selenium tissular concentrations and total mercury body burden in 6 Steller sea lion pups from the Aleutian Islands. Mar Pollut Bull. 82:175–82. 10.1016/j.marpolbul.2014.02.022. 24661459 PMC4123997

[bib51] Crawford K, McDonald RA, Bearhop S. 2008. Applications of stable isotope techniques to the ecology of mammals. Mamm Rev. 38:87–107. 10.1111/j.1365-2907.2008.00120.x.

[bib52] Crowell MG, Rahmat S, Koretsky I. 2020. Correlation of bone density in semi-aquatic and aquatic animals with ecological and dietary specializations. FASEB J. 34:1–1. 10.1096/fasebj.2020.34.s1.01860.

[bib53] Daugaard-Petersen T, Langebæk R, Rigét FF, Dyck M, Letcher RJ, Hyldstrup L, Jensen JEB, Dietz R, Sonne C. 2018. Persistent organic pollutants and penile bone mineral density in East Greenland and Canadian polar bears (*Ursus maritimus*) during 1996–2015. Environ Int. 114:212–8. 10.1016/j.envint.2018.02.022. 29522985

[bib54] Daume HV, Herr H, Mallison H, Glaubrecht M, Kaiser TM. 2023. Osteo-pathological analysis provides evidence for a survived historical ship strike in a Southern Hemisphere fin whale (*Balaenoptera physalus*). PLoS One. 18:e0281316 10.1371/journal.pone.0281316. 36812193 PMC9946264

[bib55] Davis RW . 2019a. Return to the sea: the evolution of marine mammals. In: Marine mammals. Cham: Springer International Publishing. p.7–27. 10.1007/978-3-319-98280-9.

[bib56] Davis RW . 2019b. Marine mammals adaptations for an aquatic life, marine mammals: adaptations for an aquatic life. Cham: Springer International Publishing. 10.1007/978-3-319-98280-9.

[bib57] Dawson TP, Jackson ST, House JI, Prentice IC, Mace GM. 2011. Beyond predictions: biodiversity conservation in a changing climate. Science. 332:53–8. 10.1126/science.1200303. 21454781

[bib58] de Buffrénil V, Canoville A, D’Anastasio R, Domning DP. 2010. Evolution of sirenian pachyosteosclerosis, a model-case for the study of bone structure in aquatic tetrapods. J Mamm Evol. 17:101–20.

[bib59] Dierickx K, Kersten O, van den Hurk Y, Frasier BA, Sabin R, Star B, Barrett JH. 2025. Ontogenetic changes and sexual dimorphism in the cranium and mandible of the Atlantic walrus (*Odobenus rosmarus rosmarus* L.). Anat Rec. 309:1875–903. 10.1002/ar.70050. PMC13251751 40937603

[bib60] Domning DP, De Buffrenil V. 1991. Hydrostasis in the Sirenia: quantitative data and functional interpretations. Mar Mamm Sci. 7:331–68. 10.1111/j.1748-7692.1991.tb00111.x.

[bib61] Doroftei B, Savuca A, Cretu A-M, Maftei R, Anton N, Ilea C, Doroftei M, Puha B. 2025. Microplastics and human fertility: a comprehensive review of their presence in human samples and reproductive implication. Ecotoxicol Environ Saf. 303:118939. 10.1016/j.ecoenv.2025.118939. 40876190

[bib62] Elliott Smith EA, Moss ML, Wellman HP, Gill VA, Monson DH, Newsome SD. 2025. Forecasting sea otter recolonization: insights from isotopic analysis of modern and zooarchaeological populations. Proc R Soc B Biol Sci. 292:20241682. 10.1098/rspb.2024.1682. PMC11775623 39876720

[bib63] Elliott Smith EA, Tinker MT, Whistler EL, Kennett DJ, Vellanoweth RL, Gifford-Gonzalez D, Hylkema MG, Newsome SD. 2020. Reductions in the dietary niche of southern sea otters (*Enhydra lutris nereis*) from the Holocene to the Anthropocene. Ecol Evol. 10:3318–29. 10.1002/ece3.6114. 32273989 PMC7141068

[bib64] Elmahdy YM, Orams M, Lück M, Schänzel H. 2025. Indigenous communities and marine mammal tourism management: incorporating the perspectives of the indigenous Māori people of Aotearoa/New Zealand. Front Sustain Tour. 4:1510025. 10.3389/frsut.2025.1510025.

[bib65] Evans Z, Paskulin L, Rahemtulla F, Speller CF. 2023. A comparison of minimally-invasive sampling techniques for ZooMS analysis of bone artifacts. J Archaeol Sci Rep. 47:103738. 10.1016/j.jasrep.2022.103738.

[bib66] Evershed RP, Turner-Walker G, Hedges REM, Tuross N, Leyden A. 1995. Preliminary results for the analysis of lipids in ancient bone. J Archaeol Sci. 22:277–90. 10.1006/jasc.1995.0030.

[bib67] Fay FH . 1982. Ecology and biology of the Pacific walrus, *Odobenus rosmarus divergens* illiger. North American Fauna. 74:1–266. 10.3996/nafa.74.0001.

[bib68] Feddern M, Ward E, Warlick A, Holtgrieve G. 2022. Recent divergent changes in Alaskan pinniped trophic position detected using compound-specific stable isotope analysis. Mar Ecol Prog Ser. 688:153–66. 10.3354/meps14014.

[bib69] Ferguson AW . 2020. On the role of (and threat to) natural history museums in mammal conservation: an African small mammal perspective. J Vertebr Biol. 69:20028. 10.25225/jvb.20028.

[bib70] Ferreira PG, Muñoz-Aguirre M, Reverter F, Sousa A, Amadoz A, Sodaei R, Hidalgo MR, Pervouchine D, Carbonell-Caballero J, Nurtdinov R et al. 2018. The effects of death and post-mortem cold ischemia on human tissue transcriptomes. Nat Commun. 9:490. 10.1038/s41467-017-02772-x. 29440659 PMC5811508

[bib71] Fleming AH, Kellar NM, Allen CD, Kurle CM. 2018. The utility of combining stable isotope and hormone analyses for marine megafauna research. Front Mar Sci. 5:1–15. 10.3389/fmars.2018.00338.29552559

[bib72] Foote AD, Martin MD, Louis M, Pacheco G, Robertson KM, Sinding MHS, Amaral AR, Baird RW, Baker CS, Ballance L et al. 2019. Killer whale genomes reveal a complex history of recurrent admixture and vicariance. Mol Ecol. 28:3427–44. 10.1111/mec.15099. 31131963

[bib73] Freedman V, Dorp B. 2018. Destructive sampling natural science collections: an overview for museum professionals and researchers. J Nat Sci Collections. 5:21–34.

[bib74] Fulton TL . 2012. Setting up an ancient DNA laboratory. In: Shapiro B, Hofreiter M, editors. Ancient DNA: methods and protocols. Totowa, NJ: Humana Press. p.1–11.

[bib75] Fusagawa H, Youn A, Wilkerson E, Pandya N, Feeley BT. 2024. The effects of microplastics on musculoskeletal disorder; A narrative review. Curr Rev Musculoskelet Med. 18:39–47. 10.1007/s12178-024-09932-9. 39572502 PMC11775366

[bib76] Gales NJ, Bowen WD, Johnston DW, Kovacs KM, Littnan CL, Perrin WF, Reynolds JE, Thompson PM 2009. Guidelines for the treatment of marine mammals in field research. Mar Mamm Sci. 25:725–36. 10.1111/j.1748-7692.2008.00279.x.

[bib77] Garlich-miller JL, A Stewart RE, Stewart BE, D E A Hiltz AN. 1993. Comparison of mandibular with cemental growth-layer counts for ageing Atlantic walrus (*Odobenus rosmarus rosmarus*). Can J Zool. 71:163–7. 10.1139/z93-022.

[bib78] George JC, Stimmelmayr R, Suydam R, Usip S, Givens G, Sformo T, Thewissen JGM. 2016. Severe bone loss as part of the life history strategy of bowhead whales. PLoS One. 11:e0156753. 10.1371/journal.pone.0156753. 27333180 PMC4917217

[bib79] Germanov ES, Marshall AD, Bejder L, Fossi MC, Loneragan NR. 2018. Microplastics: no small problem for filter-feeding megafauna. Trends Ecol Evol. 33:227–32. 10.1016/j.tree.2018.01.005. 29422348

[bib80] Glick P, Stein BA, Edelson NA. 2011. Scanning the conservation horizon: a guide to climate change vulnerability assessment. Washington D.C.:National Wildlife Federation.

[bib81] Goertz CEC, Frasca S, Bohach GA, Cowan DF, Buck JD, French RA, De Guise S, Maratea J, Hinckley L, Ewalt D et al. 2011. *Brucella* sp. vertebral osteomyelitis with intercurrent fatal staphylococcus aureus toxigenic enteritis in a bottlenose dolphin (*Tursiops truncatus*). J Vet Diagn Invest. 23:845–51. 10.1177/1040638711407683. 21908337 PMC3774104

[bib82] Gokhman D, Lavi E, Prüfer K, Fraga MF, Riancho JA, Kelso J, Pääbo S, Meshorer E, Carmel L. 2014. Reconstructing the DNA methylation maps of the neandertal and the Denisovan. Science (1979). 344:523–7. 10.1126/science.1250368 24786081

[bib83] Graham JH, Raz S, Hel-Or H, Nevo E. 2010. Fluctuating asymmetry: methods, theory, and applications. Symmetry. 2:466–540. 10.3390/sym2020466.

[bib84] Grahl-Nielsen O, Mjaavatten O, Tvedt E. 1993. Distinguishing between different populations of Harp Seal (*Phoca groenlandica*) by chemometry of the fatty acid profiles in the Jaw Bone. Can J Fish AquatSci. 50:1400–4. 10.1139/f93-160.

[bib85] Gray NM, Kainec K, Madar S, Tomko L, Wolfe S. 2007. Sink or swim? Bone density as a mechanism for buoyancy control in early cetaceans. Anat Rec. 290:638–53. 10.1002/ar.20533. 17516430

[bib86] Grayson DK . 1984. Quantitative zooarchaeology: topics in the analysis of archaeological faunas. Orlando, FL: Academic Press.

[bib87] Gregory MR . 1996. Plastic “Scrubbers” in hand cleansers: a further (and Minor) source for marine pollution identified. Mar Pollut Bull. 32:867–71. 10.1016/S0025-326X(96)00047-1.

[bib88] Guilminot E, Lemoine G, Pelé C, Poisson L, Surbled M, Louvet I, Mevellec JY, Rémy L. 2014. Re-treatment of whale bones—How to extract degraded fats from weakened bones?. J Cult Herit. 15:128–35. 10.1016/j.culher.2013.03.008.

[bib89] Guiry EJ, Szpak P. 2020. Quality control for modern bone collagen stable carbon and nitrogen isotope measurements. Methods Ecol Evol. 11:1049–60. 10.1111/2041-210X.13433.

[bib90] Gulland FMD, Asmutis-Silvia R, Boehm J, DiGiovanni RA, Goertz CEC, Huggins JL, Lovewell GN, Moore KM, West K. 2025. Marine mammal stranding networks in the 21st Century: whence and whither?. Mar Mamm Sci. 41:e70016. 10.1111/mms.70016.

[bib91] Hao X, Shan H, Wu C, Zhang D, Chen B. 2020. Two Decades’ variation of trace elements in bones of the endangered east asian finless porpoise (*Neophocaena asaeorientalis sunameri*) from the East China Sea China. Biol Trace Elem Res. 198:493–504. 10.1007/s12011-020-02080-4. 32080791

[bib92] Hernando-Herraez I, Prado-Martinez J, Garg P, Fernandez-Callejo M, Heyn H, Hvilsom C, Navarro A, Esteller M, Sharp AJ, Marques-Bonet T. 2013. Dynamics of DNA methylation in recent human and great ape evolution. PLoS Genet. 9:e1003763. 10.1371/journal.pgen.1003763. 24039605 PMC3764194

[bib93] Heyning J, Lento G. 2009. The evolution of marine mammals. In: Hoelzel AR, editor. Marine mammal biology: an evolutionary approach. Oxford, UK: John Wiley & Sons.

[bib94] Higgs ND, Little CTS, Glover AG. 2011. Bones as biofuel: a review of whale bone composition with implications for deep-sea biology and palaeoanthropology. Proc R Soc B Biol Sci. 278:9–17. 10.1098/rspb.2010.1267. PMC2992730 20702457

[bib95] Honda K, Fujise Y, Tatsukawa R, Itano K, Miyazaki N. 1986. Age-related accumulation of heavy metals in bone of the striped dolphin, *Stenella coeruleoalba*. Mar Environ Res. 20:143–60. 10.1016/0141-1136(86)90045-0.

[bib96] Honda K, Fujise Y, Tatsukawa R, Miyazaki N. 1984. Composition of chemical components in bone of striped dolphin, *Stenella Coeruleoalba*: distribution characteristics of major inorganic and organic components in various bones, and their age-related changes. Agric Biol Chem. 48:409–18.

[bib97] Hudson JM, Matthews CJD, Watt CA. 2021. Detection of steroid and thyroid hormones in mammlian teeth. Conserv Physiol. 9:1–15. 10.1093/conphys/coab087. PMC8633673 36439380

[bib98] Hufthammer AK, Arntsen L, Kitchener AC, Buckley M. 2018. Grey whale (*Eschrichtius robustus*) in Norwegian waters 2000 years ago. Palaeogeogr Palaeoclimatol Palaeoecol. 495:42–7. 10.1016/j.palaeo.2017.12.009.

[bib99] IPCC . 2022. Summary for policymakers. In: Pörtner H-O, Roberts DC, Poloczanska ES, Mintenbeck K, Tignor M, Alegría A, Craig M, Langsdorf S, Löschke S, Möller V, Okem A, editors. Climate change 2022: impacts, adaptation, and vulnerability. Switzerland:Cambridge University Press.

[bib100] Jackson JA, Patenaude NJ, Carroll EL, Baker CS. 2008. How few whales were there after whaling? Inference from contemporary mtDNA diversity. Mol Ecol. 17:236–51. 10.1111/j.1365-294X.2007.03497.x. 17892467

[bib101] James BD, Guerin P, Iverson Z, Allen JB. 2020. Mineralized DNA-collagen complex-based biomaterials for bone tissue engineering. Int J Biol Macromol. 161:1127–39. 10.1016/j.ijbiomac.2020.06.126. 32561285 PMC7494536

[bib102] Johnson DL, Eisaguirre JM, Taylor RL, Garlich-Miller JL. 2024. Assessing the population consequences of disturbance and climate change for the Pacific walrus. Mar Ecol Prog Ser. 740:193–211. 10.3354/meps14635.

[bib103] Jørkov MLS, Heinemeier J, Lynnerup N. 2007. Evaluating bone collagen extraction methods for stable isotope analysis in dietary studies. J Archaeol Sci. 34:1824–9.

[bib104] Kaufman JJ, Luo G, Siffert RS. 2007. A portable real-time ultrasonic bone densitometer. Ultrasound Med Biol. 33:1445–52. 10.1016/j.ultrasmedbio.2007.04.007. 17587486 PMC2063447

[bib105] Keenan M, Misarti N, Horstmann L, Crawford SG, O’Hara T, Rea LD, Avery JP. 2024. Total mercury concentrations in Steller sea lion bone: variability among locations and elements. Mar Pollut Bull. 203:116471. 10.1016/j.marpolbul.2024.116471. 38754323

[bib106] Kendall C, Eriksen AMH, Kontopoulos I, Collins MJ, Turner-Walker G. 2018. Diagenesis of archaeological bone and tooth. Palaeogeogr Palaeoclimatol Palaeoecol. 491:21–37. 10.1016/j.palaeo.2017.11.041.

[bib107] Khairy M, Brault E, Dickhut R, Harding KC, Harkonen T, Karlsson O, Lehnert K, Teilmann J, Lohmann R. 2021. Bioaccumulation of PCBs, OCPs and PBDEs in Marine Mammals From West Antarctica. Front Mar Sci. 8:768715. 10.3389/fmars.2021.768715.

[bib108] Kistler L, Ware R, Smith O, Collins M, Allaby RG. 2017. A new model for ancient DNA decay based on paleogenomic meta-analysis. Nucleic Acids Res. 45:6310–20. 10.1093/nar/gkx361. 28486705 PMC5499742

[bib109] Klingenberg CP, Barluenga M, Meyer A. 2002. Shape analysis of symmetric structures: quantifying variation among individuals and asymmetry. Evolution (NY). 56:1909–20. 10.1111/j.0014-3820.2002.tb00117.x 12449478

[bib110] Koskela A, Finnilä MA, Korkalainen M, Spulber S, Koponen J, Håkansson H, Tuukkanen J, Viluksela M. 2016. Effects of developmental exposure to perfluorooctanoic acid (PFOA) on long bone morphology and bone cell differentiation. Toxicol Appl Pharmacol. 301:14–21. 10.1016/j.taap.2016.04.002. 27068293

[bib111] Koskela A, Koponen J, Lehenkari P, Viluksela M, Korkalainen M, Tuukkanen J. 2017. Perfluoroalkyl substances in human bone: concentrations in bones and effects on bone cell differentiation. Sci Rep. 7:6841. 10.1038/s41598-017-07359-6. 28754927 PMC5533791

[bib112] Kuczumow A, Gorzelak M, Kosiński J, Lasota A, Blicharski T, Gągała J, Nowak J, Jarzębski M, Jabłoński M. 2022. Hierarchy of bioapatites. Int J Mol Sci. 23:9537. 10.3390/ijms23179537. 36076932 PMC9455617

[bib113] Kulus MJ, Cebulski K, Kmiecik P, Sputa-Grzegrzółka P, Grzelak J, Dąbrowski P. 2024. New equations for the estimation of the age of the formation of the Harris lines. 14:501. 10.3390/life14040501 PMC11051040 38672771

[bib114] Laeta M, Oliveira JA, Siciliano S, Lambert O, Jensen FH, Galatius A. 2023. Cranial asymmetry in odontocetes: a facilitator of sonic exploration?. Zoology. 160:126108. 10.1016/j.zool.2023.126108. 37633185

[bib115] Laws RM . 1960. Laminated structure of bones from some marine mammals. Nature. 187:338–9. 10.1038/187338a0. 14414747

[bib116] Lee HY, Hong SR, Lee JE, Hwang IK, Kim NY, Lee JM, Fleckhaus J, Jung S-E, Lee YH. 2020. Epigenetic age signatures in bones. Forensic Sci Int Genet. 46:102261. 10.1016/j.fsigen.2020.102261. 32087494

[bib117] Lee-Thorp JA . 2008. ON ISOTOPES AND OLD BONES*. Archaeometry. 50:925–50. 10.1111/j.1475-4754.2008.00441.x.

[bib118] Liebenau K, Kiel S, Vardeh D, Treude T, Thiel V. 2015. A quantitative study of the degradation of whale bone lipids: implications for the preservation of fatty acids in marine sediments. Org Geochem. 89–90:23–30. 10.1016/j.orggeochem.2015.09.005.

[bib119] Lonati GL, Howell AR, Schueller P, Deutsch CJ. 2021. Using tetracycline to evaluate age estimation in a long-lived aquatic mammal. Wildl Soc Bull. 45:340–50. 10.1002/wsb.1192.

[bib120] MacCracken JG, Benter RB. 2016. Trend in Pacific walrus (Odobenus rosmarus divergens) tusk asymmetry, 1990-2014. Mar Mamm Sci. 32:588–601. 10.1111/mms.12286.

[bib121] Manuta N, Januzi V, Ozkan E, Ünal B, Çakar B, İşbilir F, Melnyk OP, Güzel BC. 2026. Comparative fluctuating asymmetry and directional asymmetry in four cattle skulls. Vet Med Sci. 12:1–9. 10.1002/vms3.70796. PMC12812312 41548210

[bib122] Marsh H, Ahuanari L, del Aguila V, Haami B, Laureano M, Loban F, Tarriasuk Q, Vozhikov II, Belonovich OA, Kendall S et al. 2022. Elders’ voices: examples of contemporary indigenous knowledge of marine mammals. In: Notarbartolo di Sciara G, Würsig B, editors. Marine mammals: the evolving human factor. Cham: Springer International Publishing. p.337–74.

[bib123] Martinoia V, Craig OE, Charlton S, Britton K, Sheridan A, Bones A, Talbot H, MacDonald R, Richards M. 2025. High-resolution compound-specific δ15N isotope dietary study of humans from the Scottish Mesolithic and Neolithic. Archaeometry. 67:1309–26. 10.1111/arcm.13089.

[bib124] Matthews CJD, Ferguson SH. 2014. Spatial segregation and similar trophic-level diet among eastern Canadian Arctic/north-west Atlantic killer whales inferred from bulk and compound specific isotopic analysis. J Mar Biol Assoc UK. 94:1343–55. 10.1017/S0025315413001379.

[bib125] Matthews CJD, Ruiz-Cooley RI, Pomerleau C, Ferguson SH. 2020. Amino acid δ15N underestimation of cetacean trophic positions highlights limited understanding of isotopic fractionation in higher marine consumers. Ecol Evol. 10:3450–62. 10.1002/ece3.6142. 32274001 PMC7141024

[bib126] McGrath K, Montes TAK da S, Fossile T, da Rocha Bandeira D, Borba FM, Cremer MJ, van der Sluis LG, Higham T, Klahold Rosa AP, Saña M et al. 2026. Molecular and zooarchaeological identification of 5000 year old whale-bone harpoons in coastal Brazil. Nat Commun. 17. 10.1038/s41467-025-67530-w. PMC12789592 41513657

[bib127] Merrill GB, Hermabessiere L, Rochman CM, Nowacek DP 2023. Microplastics in marine mammal blubber, melon, & other tissues: evidence of translocation. Environ Pollut. 335:122252. 10.1016/j.envpol.2023.122252. 37541381

[bib128] Metcalf V . 2024. People and walrus on St. Lawrence Island, Alaska. Environment. 66:50–3.

[bib129] Metcalf V, Apassingok D, Campbell I, Gologergen A, Gologergen B, Noongwook E, Huntington HP, Garlich-Miller JL, Johnson DL. 2025. Co-producing knowledge about the Pacific walrus and climate change. Arct Sci. 11:1–13. 10.1139/as-2024-0061.

[bib130] Mills KK, Hildebrandt KPB, Everson KM, Horstmann L, Misarti N, Olson LE. 2024. Ancient DNA indicates a century of overhunting did not reduce genetic diversity in Pacific Walruses (*Odobenus rosmarus divergens*). Sci Rep. 14:8257. 10.1038/s41598-024-57414-2. 38589385 PMC11001934

[bib131] Misarti N, Finney BP, Maschner H. 2011. Reconstructing site organization in the eastern Aleutian Islands, Alaska using multi-element chemical analysis of soils. J Archaeol Sci. 38:1441–55. 10.1016/j.jas.2011.02.007.

[bib132] Misarti N, Finney B, Maschner H, Wooller MJ. 2009. Changes in northeast Pacific marine ecosystems over the last 4500 years: evidence from stable isotope analysis of bone collagen from archeological middens. Holocene. 19:1139–51. 10.1177/0959683609345075.

[bib133] Moore MJ, Early GA. 2004. Cumulative sperm whale bone damage and the bends. Science (1979). 306:2215. 10.1126/science.1105452 15618509

[bib134] Moorhead A . 2022. Accumulation of persistent organic pollutants in marine mammals: a case study on cetaceans, pinnipeds, and sirenians (Master of Science).

[bib135] Møller AP . 1997. Developmental stability and fitness: a review. Am Nat. 149:916–32. 10.1086/286030. 18811255

[bib136] National Museum of Natural History . 2026. Destructive analysis Smithsonian. https://naturalhistory.si.edu/research/vertebrate-zoology/mammals/collections-access/destructive-analysis (16 February 2026, date last accessed).

[bib137] Newsome SD, Koch PL, Etnier MA, Aurioles-Gamboa D . 2006. Using carbon and nitrogen isotope values to investigate maternal strategies in Northeast Pacific otariids. Mar Mamm Sci. 22:556–72. 10.1111/j.1748-7692.2006.00043.x.

[bib138] Newsome SD, Clementz MT, Koch PL. 2010. Using stable isotope biogeochemistry to study marine mammal ecology. Mar Mammal Sci. 26:509–72.

[bib139] Nganvongpanit K, Soponteerakul R, Kaewkumpai P, Punyapornwithaya V, Buddhachat K, Nomsiri R, Kaewmong P, Kittiwatanawong K, Chawangwongsanukun R, Angkawanish T et al. 2017. Osteoarthritis in two marine mammals and 22 land mammals: learning from skeletal remains. J Anat. 231:140–55. 10.1111/joa.12620. 28542897 PMC5472524

[bib140] Norris D . 1997. Vertebrate endocrinology. 3rd ed. San Diego, CA: Academic Press.

[bib141] Norris K, Tweddle J. 2026. How can natural history museums help nature recover?. Trends Ecol Evol. 41:309–17. 10.1016/j.tree.2025.12.008. 41486049

[bib142] Núñez-Nogueira G, Pérez-López A, JM S-C. 2019. As, Cr, Hg, Pb, and Cd concentrations and bioaccumulation in the Dugong *Dugong dugon* and Manatee *Trichechus manatus*: a review of body burdens and distribution. Int J Environ Res Public Health. 16:404. 10.3390/ijerph16030404. 30708981 PMC6388294

[bib143] O’Connor J, Horstmann L, Chenoweth E, VonDuyke A, Stimmelmayr R. 2026. Assessment of microplastics exposure, translocation, and potential long-term storage in Alaska polar bears (*Ursus maritumus*). Poster Presentation at the Alaska Marine Science Symposium. Anchorage, AK. January 26, 2026.

[bib144] Pálsdóttir AH, Bläuer A, Rannamäe E, Boessenkool S, Hallsson JH. 2019Not a limitless resource: ethics and guidelines for destructive sampling of archaeofaunal remains. R Soc Open Sci. 6:191059. 10.1098/rsos.191059. 31824712 PMC6837180

[bib145] Pedersen JS, Valen E, Velazquez AMV, Parker BJ, Rasmussen M, Lindgreen S, Lilje B, Tobin DJ, Kelly TK, Vang S et al. 2014. Genome-wide nucleosome map and cytosine methylation levels of an ancient human genome. Genome Res. 24:454–66. 10.1101/gr.163592.113. 24299735 PMC3941110

[bib146] Pemmer B, Roschger A, Wastl A, Hofstaetter JG, Wobrauschek P, Simon R, Thaler HW, Roschger P, Klaushofer K, Streli C. 2013. Spatial distribution of the trace elements zinc, strontium and lead in human bone tissue. Bone. 57:184–93. 10.1016/j.bone.2013.07.038. 23932972 PMC3807669

[bib147] Pérez F, Nadal M, Navarro-Ortega A, Fàbrega F, Domingo JL, Barceló D, Farré M. 2013. Accumulation of perfluoroalkyl substances in human tissues. Environ Int. 59:354–62. 23892228 10.1016/j.envint.2013.06.004

[bib148] Petitguyot MAC, Fariñas-Bermejo A, Brownlow A, Ahola MP, Álvarez Neche, Arbelo M, Authier M, Balsera Riesgo R, Berrow S, Bjørge A et al. 2025. European stranding networks as a tool for monitoring marine mammal populations (Part I): towards optimising the functioning of networks. ICES J Mar Sci. 82. 10.1093/icesjms/fsaf194.

[bib149] Pichler FB, Dalebout ML, Baker CS. 2001. Nondestructive DNA extraction from sperm whale teeth and scrimshaw. Mol Ecol Notes. 1:106–9. 10.1046/j.1471-8278.2001.00027.x.

[bib150] Polanowski AM, Robbins J, Chandler D, Jarman SN. 2014. Epigenetic estimation of age in humpback whales. Mol Ecol Resour. 14:976–87. 10.1111/1755-0998.12247. 24606053 PMC4314680

[bib151] Pollard AM, Heron C, Armitage RA. 2020. Archaeological chemistry Royal Society of Chemistry. 3rd ed. Cambridge, England: Royal Society of Chemistry.

[bib152] Powell JWB, Duffield DA, Kaufman JJ, Luo G, Lovewell GN, McFee WE. 2021. Bone mineral density of the common bottlenose dolphin radius: a primary skeletal site for clinical bone densitometry and preliminary descriptive data set using archival specimens. Mar Mamm Sci. 37:374–84. 10.1111/mms.12769.

[bib153] Pozzi CM, Ladio AH. 2023. Variation of local zoological knowledge about Southern river otter and other semi-aquatic mammals in Nahuel Huapi National Park (Argentina). J Ethnobiol Ethnomed. 19. 10.1186/s13002-023-00590-8. PMC10170775 37161447

[bib154] Prajapati A, Narayan Vaidya A, Kumar AR. 2022. Microplastic properties and their interaction with hydrophobic organic contaminants: a review. Environ Sci Pollut Res. 29:49490–512. 10.1007/s11356-022-20723-y. 35589887

[bib155] Quinn RL, Beasley MM, Gocha TP, Mavroudas SR. 2025. Differential human bone remodeling rates and implications for the temporal resolution of geoprofiling isotopes. Forensic Sci Int. 370:112454. 10.1016/j.forsciint.2025.112454. 40168831

[bib156] Read FL, Hohn AA, Lockyer CH. 2018. A review of age estimation methods in marine mammals with special reference to monodontids. NAMMCO Sci Publ. 10:1–67. 10.7557/3.4474.

[bib157] Rendina-Ruedy E, Rosen CJ. 2020. Lipids in the bone marrow: an evolving perspective. Cell Metab. 31:219–31. 10.1016/j.cmet.2019.09.015. 31668874 PMC7004849

[bib158] Riofrío-Lazo M, Aurioles-Gamboa D. 2013. Timing of isotopic integration in marine mammal skull: comparative study between calcified tissues. Rapid Commun Mass Spectrom. 27:1076–82. 10.1002/rcm.6556. 23592211

[bib159] Robinson AL, Elliott Smith EA, Besser AC, Newsome SD. 2024. Tissue-specific carbon isotope patterns of amino acids in southern sea otters. Oecologia. 204:13–24. 10.1007/s00442-023-05505-8. 38227253

[bib160] Rochman CM, Kurobe T, Flores I, Teh SJ. 2014. Early warning signs of endocrine disruption in adult fish from the ingestion of polyethylene with and without sorbed chemical pollutants from the marine environment. Sci Total Environ. 493:656–61. 10.1016/j.scitotenv.2014.06.051. 24995635

[bib161] Rohland N, Hofreiter M. 2007. Ancient DNA extraction from bones and teeth. Nat Protoc. 2:1756–62. 10.1038/nprot.2007.247. 17641642

[bib162] Rohland N, Siedel H, Hofreiter M. 2010. A rapid column-based ancient DNA extraction method for increased sample throughput. Mol Ecol Resour. 10:677–83. 10.1111/j.1755-0998.2009.02824.x. 21565072

[bib163] Rosel PE, Taylor BL, Hancock-Hanser BL, Morin PA, Archer FI, Lang AR, Mesnick SL, Pease VL, Perrin WF, Robertson KM et al. 2017. A review of molecular genetic markers and analytical approaches that have been used for delimiting marine mammal subspecies and species. Mar Mamm Sci. 33:56–75. 10.1111/mms.12412.

[bib164] Rosenbaum HC, Egan MG, Clapham PJ, Brownell RL, DeSalle R. 1997. An effective method for isolating DNA from historical specimens of baleen. Mol Ecol. 6:677–81. 10.1046/j.1365-294X.1997.00230.x. 9226948

[bib165] Roze D, Rousse F, Michalakis Y. 2005. Germline bottlenecks, biparental inheritance and selection on mitochondrial variants: a two-level selection model. Genetics. 170:1385–99. 10.1534/genetics.104.039495. 15911581 PMC1451199

[bib166] Rusu I, Paica I, Vulpoi A, Radu C, Mircea C, Dobrinescu C, Bodolică V, Kelemen B. 2019. Dual DNA-protein extraction from human archeological remains. Archaeol Anthropol Sci. 11:3299–307. 10.1007/s12520-018-0760-1.

[bib167] Rüther PL, Husic IM, Bangsgaard P, Gregersen KM, Pantmann P, Carvalho M, Godinho RM, Friedl L, Cascalheira J, Taurozzi AJ et al. 2022. SPIN enables high throughput species identification of archaeological bone by proteomics. Nat Commun. 13:2458. 10.1038/s41467-022-30097-x. 35513387 PMC9072323

[bib168] Saeki K, Nakajima M, Noda K, Loughlin TR, Baba N, Kiyota M, Tatsukawa R, Calkins DG. 1999. Vanadium accumulation in pinnipeds. Arch Environ Contam Toxicol. 36:81–6. 10.1007/s002449900445. 9828265

[bib169] Salvador RB, Cunha CM. 2020. Natural history collections and the future legacy of ecological research. Oecologia. 192:641–6. 10.1007/s00442-020-04620-0. 32056020

[bib170] Sánchez-Chardi A, García-Pando M, MJ L-F. 2013. Chronic exposure to environmental stressors induces fluctuating asymmetry in shrews inhabiting protected mediterranean sites. Chemosphere. 93:916–23. 10.1016/j.chemosphere.2013.05.056. 23800592

[bib171] San Martín AA, Macnie SV, Goodall RNP, Boy CC. 2016. Pathology in skeletons of Peale’s dolphin *Lagenorhynchus australis* from southern South America. Dis Aquat Organ. 120:9–15. 27304866 10.3354/dao03010

[bib172] Santos LHMLM, Insa S, Arxé M, Buttiglieri G, Rodríguez-Mozaz S, Barceló D. 2023. Analysis of microplastics in the environment: identification and quantification of trace levels of common types of plastic polymers using pyrolysis-GC/MS. MethodsX. 10:102143. 10.1016/j.mex.2023.102143. 37007617 PMC10050779

[bib173] Schandorff S . 1997. Developmental stability and skull lesions in the harbour seal (*Phoca vitulina*) in the 19th and 20th Centuries. Ann Zool Fennici. 34:151–66.

[bib174] Schantz MM, Koster BJ, Wise SA, Becker PR. 1993. Determination of PCBs and chlorinated hydrocarbons in marine mammal tissues. Sci Total Environ. 139:323–45. 10.1016/0048-9697(93)90031-Z. 8272838

[bib175] Schoeman RP, Patterson-Abrolat C, Plön S. 2020. A global review of vessel collisions with marine animals. Front Mar Sci. 7. 10.3389/fmars.2020.00292.

[bib176] Schrader S, Brewster KAJ, Hall R, Giera M, Sánchez-López E. 2026. Paleohormones: a new biomolecular perspective on archaeological bone. Heliyon. 12:e44312. 10.1016/j.heliyon.2025.e44312.

[bib177] Scott M . 2021. Chew the Fat: an examination of the preservation of fatty acids in archaeological bone [M. A. Thesis]. Peterborough, Ontario, Canada: Trent University.

[bib178] Séon N, Amiot R, Suan G, Lécuyer C, Fourel F, Vinçon-Laugier A, Charbonnier S, Vincent P. 2024. Regional heterothermies recorded in the oxygen isotope composition of harbour seal skeletal elements. J Therm Biol. 120:103825. 38430855 10.1016/j.jtherbio.2024.103825

[bib179] Sharma V, Srinivasan A, Nikolajeff F, Kumar S. 2021. Biomineralization process in hard tissues: the interaction complexity within protein and inorganic counterparts. Acta Biomater. 120:20–37. 10.1016/j.actbio.2020.04.049 32413577

[bib180] Simokon MV, Trukhin AM. 2021. Analysis of essential and non-essential trace elements in the organs of a mother–fetus pair of spotted seals (*Phoca largha*) from the Sea of Japan. Environ Sci Pollut Res. 28:60622–34. 10.1007/s11356-021-14971-7. 34164788

[bib181] Simpson R, Cooper DML, Swanston T, Coulthard I, Varney TL. 2021. Historical overview and new directions in bioarchaeological trace element analysis: a review. Archaeol Anthropol Sci. 13:1–27. 10.1007/s12520-020-01262-4. PMC7810633 33520004

[bib182] Sletten A, Bryan A, Iken K, Olnes J, Horstmann L. 2025. Microplastics in spotted seal stomachs from the Bering and Chukchi seas in 2012 and 2020. Mar Pollut Bull. 214:117770 10.1016/j.marpolbul.2025.117770. 40024193

[bib183] Smith KJ, Mead JG, Peterson MJ. 2021a. Specimens of opportunity provide vital information for research and conservation regarding elusive whale species. Environ Conserv. 48:84–92. 10.1017/S0376892920000521.

[bib184] Smith KJ, Sparks JP, Timmons ZL, Peterson MJ. 2020. Cetacean skeletons demonstrate ecologically relevant variation in intraskeletal stable isotopic values. Front Mar Sci. 7:1–9. 10.3389/fmars.2020.00388.32802822

[bib185] Smith KJ, Trueman CN, France CAM, Sparks JP, Brownlow AC, Dähne M, Davison NJ, Guðmundsson G, Khidas K, Kitchener AC et al. 2021b. Stable isotope analysis of specimens of opportunity reveals ocean-scale site fidelity in an elusive whale species. Front Conserv Sci. 2:13. 10.3389/fcosc.2021.653766.

[bib186] Soga M, Gaston KJ. 2018. Shifting baseline syndrome: causes, consequences, and implications. Front Ecol Environ. 16:222–30. 10.1002/fee.1794.

[bib187] Sonne C, Dietz R, Born EW, Riget FF, Kirkegaard M, Hyldstrup L, Letcher RJ, Muir DCG. 2004. Is bone mineral composition disrupted by organochlorines in East Greenland polar bears (*Ursus maritimus*)?. Environ Health Perspect. 112:1711–6. 10.1289/ehp.7293. 15579418 PMC1253664

[bib188] Sonne C, Siebert U, Gonnsen K, Desforges J-P, Eulaers I, Persson S, Roos A, Bäcklin B-M, Kauhala K, Tange Olsen M et al. 2020. Health effects from contaminant exposure in Baltic Sea birds and marine mammals: a review. Environ Int. 139:105725. 10.1016/j.envint.2020.105725. 32311628

[bib189] Stevens RE, Pederzani S, Britton K, Wexler SK. 2025. Bones and teeth isotopes as archives for palaeoclimatic, palaeoenvironmental and palaeoecological data. Quat Sci Rev. 357:109320. 10.1016/j.quascirev.2025.109320.

[bib190] Stock F, Kochleus C, Bänsch-Baltruschat B, Brennholt N, Reifferscheid G. 2019. Sampling techniques and preparation methods for microplastic analyses in the aquatic environment—a review. TrAC Trends Anal Chem. 113:84–92. 10.1016/j.trac.2019.01.014.

[bib191] Stott AW, Davies E, Evershed RP, Tuross N. 1997. Monitoring the routing of dietary and biosynthesised lipids through compound—specific stable isotope (δ 13 C) measurements at natural abundance. Naturwissenschaften. 84:82–6. 10.1007/s001140050354. 9121591

[bib192] Stuart-Smith SJ, Jepson PD. 2017. Persistent threats need persistent counteraction: responding to PCB pollution in marine mammals. Mar Policy. 84:69–75. 10.1016/j.marpol.2017.06.033.

[bib193] Styring AK, Sealy JC, Evershed RP. 2010. Resolving the bulk δ15N values of ancient human and animal bone collagen via compound-specific nitrogen isotope analysis of constituent amino acids. Geochim Cosmochim Acta. 74:241–51. 10.1016/j.gca.2009.09.022.

[bib194] Suarez A, Tsutsui N. 2004. The value of museum collections for research and society. Bioscience. 54:66–74. 10.1641/0006-3568(2004)054[0066:TVOMCF]2.0.CO;2.

[bib195] Suarez-Bregua P, Guerreiro PM, Rotllant J. 2018. Stress, glucocorticoids and bone: a review from mammals and fish. Front Endocrinol (Lausanne). 9:526. 10.3389/fendo.2018.00526. 30250453 PMC6139303

[bib196] Sun D, Zhou X, Yu Z, Xu S, Seim I, Yang G. 2019. Accelerated evolution and diversifying selection drove the adaptation of cetacean bone microstructure. BMC Evol Biol. 19. 10.1186/s12862-019-1509-x. PMC6813995 31651232

[bib197] Swaddle JP, Witter MS. 1994. Food, feathers and fluctuating asymmetries. Proc R Soc Lond B Biol Sci. 255:147–52.

[bib198] Szpak P, Buckley M. 2020. Sulfur isotopes (δ34S) in Arctic marine mammals: indicators of benthic vs. pelagic foraging. Mar Ecol Prog Ser. 653:205–16. 10.3354/meps13493.

[bib199] Szpak P, Buckley M, Darwent CM, Richards MP. 2018. Long-term ecological changes in marine mammals driven by recent warming in northwestern Alaska. Glob Chang Biol. 24:490–503. 10.1111/gcb.13880. 28850766

[bib200] Tanabe S, Tatsukawa R, Tanaka H, Maruyama K, Miyazaki N, Fujiyama T. 1981. Distribution and Total Burdens of Chlorinated Hydrocarbons in Bodies of Striped Dolphins (*Stenella coeruleoalba*). Agric Biol Chem. 45:2569–78. 10.1080/00021369.1981.10864933.

[bib201] Tansel B . 2026. Trophic-level accumulation and transfer of legacy and emerging contaminants in marine biota: meta-analysis of mercury, PCBs, microplastics, PFAS, PAHs. Mar Pollut Bull. 222:118666. 10.1016/j.marpolbul.2025.118666. 40915019

[bib202] Taylor N, Clark CT, Misarti N, Horstmann L. 2020. Determining sex of adult Pacific walruses from mandible measurements. J Mammal. 101:941–50. 10.1093/jmammal/gyaa051. 33033468 PMC7528639

[bib203] Teixeira CR, Troina GC, Daura-Jorge FG, Simões-Lopes PC, Botta S. 2022. A practical guide on stable isotope analysis for cetacean research. Mar Mamm Sci. 38:1200–28. 10.1111/mms.12911.

[bib204] Thurman LL, Stein BA, Beever EA, Foden W, Geange SR, Green N, Gross JE, Lawrence DJ, LeDee O, Olden JD et al. 2020. Persist in place or shift in space? Evaluating the adaptive capacity of species to climate change. Front Ecol Environ. 18:520–8. 10.1002/fee.2253.

[bib205] Tintut Y, Demer LL. 2014. Effects of bioactive lipids and lipoproteins on bone. Trends Endocrinol Metab. 25:53–9. 10.1016/j.tem.2013.10.001. 24183940 PMC3946677

[bib206] Tomlinson RE, Silva MJ. 2013. Skeletal blood flow in bone repair and maintenance. Bone Res. 1:311–22. 10.4248/BR201304002. 26273509 PMC4472118

[bib207] Tont SA, Pearcy WG, Arnold JS. 1977. Bone structure of some marine vertebrates. Mar Biol. 39:191–6. 10.1007/BF00387004.

[bib208] Tsutaya T, Gakuhari T, Asahara A, Yoneda M. 2017. Isotopic comparison of gelatin extracted from bone powder with that from bone chunk and development of a framework for comparison of different extraction methods. J Archaeol Sci Rep. 11:99–105.

[bib209] Turnbull BS, Cowan DF. 1999. Synovial joint disease in wild cetaceans. J Wildl Dis. 35:511–8. 10.7589/0090-3558-35.3.511. 10479085

[bib210] Unger CA, Hope MC, Aladhami AK, Cotham WE, Socia CE, Rice BC, Clegg DJ, Velázquez KT, LaVoie HA, Hollis F et al. 2023. A novel tissue-specific insight into sex steroid fluctuations throughout the murine estrous cycle. Endocrinology. 165:bqad175 10.1210/endocr/bqad175. 37967240 PMC11032246

[bib211] Usha K, Nandeesh B. 2012. Physiology of bone formation, remodeling, and metabolism. In: Fogelman I, Gnanasegaran G, Van der Wall H, editors. Radionuclide and hybrid bone imaging. Berlin and Hedelberg: Springer-Verlag. p. 29–57.

[bib212] Vainberg A, Abakumov E. 2025. Microplastic exposure for pinnipeds (*Pinnipedia*): a rapid review. Ecologies. 6:26. 10.3390/ecologies6020026.

[bib213] van Bressem M, van Waerebeek K, Montes D, Kennedy S, Reyes J, Garcia-Godos I, Onton-Silva K, Alfaro-Shigueto J. 2006. Diseases, lesions and malformations in the long-beaked common dolphin *Delphinus capensis* from the Southeast Pacific. Dis Aquat Organ. 68:149–65. 10.3354/dao068149. 16532606

[bib214] Vaye O, Ngumbu RS, Xia D. 2022. A review of the application of comprehensive two-dimensional gas chromatography MS-based techniques for the analysis of persistent organic pollutants and ultra-trace level of organic pollutants in environmental samples. Rev Anal Chem. 41:63–73. 10.1515/revac-2022-0034.

[bib215] Vighi M, Borrell A, Aguilar A. 2017. Bone as a surrogate tissue to monitor metals in baleen whales. Chemosphere. 171:81–8. 10.1016/j.chemosphere.2016.12.036. 28011406

[bib216] Wandeler P, Hoeck PEA, Keller LF. 2007. Back to the future: museum specimens in population genetics. Trends Ecol Evol. 22:634–42. 10.1016/j.tree.2007.08.017. 17988758

[bib217] Watanabe I, Ichihashi H, Tanabe S, Amano M, Miyazaki N, Petrovc EA, Tatsukawaax R. 1996. Trace element accumulation in Baikal seal (*Phoca sibirica*) from the Lake Baikal. Environ Pollut. 94:169–79. 10.1016/S0269-7491(96)00079-6. 15093503

[bib218] Wawrzyniak A, Balawender K. 2022. Structural and metabolic changes in bone. Animals. 12:1946. 35953935 10.3390/ani12151946 PMC9367262

[bib219] Welker F, Collins MJ, Thomas JA, Wadsley M, Brace S, Cappellini E, Turvey ST, Reguero M, Gelfo JN, Kramarz A et al. 2015. Ancient proteins resolve the evolutionary history of Darwin’s South American ungulates. Nature. 522:81–4. 10.1038/nature14249. 25799987

[bib220] Wellman HP, Zhang H, Yang DY, Evans ZD, Miner M, Speller CF. 2025. Native American use of cetaceans in pre-contact Oregon: biomolecular and taphonomic analyses illuminate human–cetacean relationships. J Island Coastal Archaeol. 20:472–95. 10.1080/15564894.2023.2255582.

[bib221] Whiteman JP, Elliott Smith EA, Besser AC, Newsome SD. 2019. A Guide to Using Compound-Specific Stable Isotope Analysis to Study the Fates of Molecules in Organisms and Ecosystems. Diversity (Basel). 11:8. 10.3390/d11010008.

[bib222] Winfield ZC, Mansouri F, Potter CW, Sabin R, Trumble SJ, Usenko S. 2020. Eighty years of chemical exposure profiles of persistent organic pollutants reconstructed through baleen whale earplugs. Sci Total Environ. 737:139564. 10.1016/j.scitotenv.2020.139564. 32512296

[bib223] Yamamoto Y, Honda K, Hidaka H, Tatsukawa R. 1987. Tissue distribution of heavy metals in Weddell seals (*Leptonychotes weddellii*). Mar Pollut Bull. 18:164–9. 10.1016/0025-326X(87)90240-2.

[bib224] Yamoah AKK, Harland J, McLaughlin R, Talbot HM, Fontanals-Coll M, Craig OE, Orton D. 2025. Investigating long-term trophic stability in North Atlantic cod (*Gadus morhua*) through nitrogen stable isotope analysis of amino acids. Phil Trans R Soc B Biol Sci. 380:20240028. 10.1098/rstb.2024.0028. PMC12242297 40635432

[bib225] Yang Q, Peng Y, Wu X, Cao X, Zhang P, Liang Z, Zhang J, Zhang Y, Gao P, Fu Y et al. 2025. Microplastics in human skeletal tissues: presence, distribution and health implications. Environ Int. 196:109316. 10.1016/j.envint.2025.109316. 39946929

[bib226] Yarrow JF, Conover CF, Lipinska JA, Santillana CA, Wronski TJ, Borst SE. 2010. Methods to quantify sex steroid hormones in bone: applications to the study of androgen ablation and administration. Am J Physiol Endocrinol Metab. 299:E841–47. 10.1152/ajpendo.00384.2010. 20739509

[bib227] Zantis LJ, Carroll EL, Nelms SE, Bosker T. 2021. Marine mammals and microplastics: a systematic review and call for standardisation. Environ Pollut. 269:116142. 10.1016/j.envpol.2020.116142. 33288297

